# Supplementary material for: Active transcription and epigenetic reactions synergistically regulate meso-scale genomic organization
Source: Nat Commun. 2024 May 21;15:4338. doi: 10.1038/s41467-024-48698-z (PMC11109243; doi:10.1038/s41467-024-48698-z)
Supplement: Supplementary file 1 — Supplementary Information [file 41467_2024_48698_MOESM1_ESM.pdf]

SUPPLEMENTARY INFORMATION

Active Transcription and Epigenetic Reactions Synergistically Regulate Meso-Scale Genomic Organization

Aayush Kant [1,2], Zixian Guo [1,3], Vinayak Vinayak [1,2], Maria Victoria Neguembor [4], Wing Shun Li [5,6], Vasundhara Agrawal [6,7], Emily Pujadas [6], Luay Almassalha [6,8], Vadim Backman [6,7], Melike Lakadamyali [1,9], Maria Pia Cosma [4,10,11], Vivek B. Shenoy [1,2,3]

[1] Center for Engineering Mechanobiology, University of Pennsylvania, Philadelphia, PA, 19104, USA

[2] Department of Materials Science and Engineering, University of Pennsylvania, Philadelphia, PA, 19104, USA

[3] Department of Mechanical Engineering and Applied Mechanics, University of Pennsylvania, Philadelphia, PA, 19104, USA

[4] Centre for Genomic Regulation (CRG), The Barcelona Institute of Science and Technology, 08003 Barcelona, Spain

[5] Department of Applied Physics, Northwestern University, Evanston, IL 60208, USA

[6] Center for Physical Genomics and Engineering, Northwestern University, Evanston, IL 60202, USA

[7] Department of Biomedical Engineering, Northwestern University, Evanston, IL 60208, USA

[8] Department of Gastroenterology and Hepatology, Northwestern Memorial Hospital, Chicago, IL 60611, USA

[9] Department of Physiology, Perelman School of Medicine, University of Pennsylvania, Philadelphia, PA 19104, USA

[10] ICREA, Barcelona, 08010, Spain

[11] Universitat Pompeu Fabra (UPF), Barcelona, 08003, Spain

Contents

S1 Extended Methods 2
S1.1 Mathematical description of chromatin distribution in nucleus........ 2
S1.2 Free energy landscape of the nucleus:........ 2
S1.3 Specific form of the energetic contribution from chromatin-chromatin interactions.... 4
S1.4 Diffusion and reaction kinetics........ 5
S1.5 Rescaling the governing equations........ 8
S1.6 Polymer analogy of the roles played by reaction and diffusion kinetics........ 9
S1.7 Neighborhood dependent reaction-kinetics........11
S1.8 Chromatin clustering of STORM images........14
S1.9 Quantitative analysis of chromatin distribution in STORM images........15
S1.10 ChromSTEM sample preparation, imaging, and reconstruction for BJ Fibroblasts...15
S1.11 Domain Center Mapping and Statistical Analysis........16
S1.12 ActD Treatment for PWS and ChromSTEM imaging........17
S1.13 PWS image acquisition and approximation of domain size scales........17
S2 Heterochromatin domain morphology dependence on Epigenetic Rates........17
S3 Theoretical analysis of average chromatin phase contents determined by reactions......18
S4 Domain size determination in presence of transcription – theoretical analysis........20
S5 A characteristic size of heterochromatin domains is not obtained without reactions.......24
S6 Stable domain radius is not significantly regulated by interfacial effects........25
S7 LAD thickness determination in presence of transcription – theoretical analysis........25
S8 Model calibration and validation........27
S9 Translating the model predictions into physical dimensions........29
S10 The qualitative predictions of the model are agnostic to extent of compaction of the heterochromatin phase........30
S11 Extending the model to incorporate multiple states of chromatin........31
REFERENCES........33

S1 Extended Methods

S1.1 Mathematical description of chromatin distribution in nucleus

To investigate the organization of chromatin in the nucleus, we develop a mathematical model for the phase separation of heterochromatin and euchromatin considering chromatin-chromatin interactions, chromatin-lamina interactions, epigenetic regulation of chromatin via histone acetylation or methylation, and the role of transcriptional regulators. We consider three nuclear constituents – nucleoplasm and chromatin in either heterochromatin or euchromatin phases.

At any point $\boldsymbol{x}$ in the nucleus, at a time t, consider an infinitesimal observation window of volume $V(\boldsymbol{x},t)$. Let the volume of nucleoplasm, euchromatin and heterochromatin within this observation window be $V_n(\boldsymbol{x},t)$, $V_e(\boldsymbol{x},t)$ and $V_h(\boldsymbol{x},t)$. Note that $V = V_n + V_e + V_h$. Thus,

$$\frac{V_n}{V} + \frac{V_e}{V} + \frac{V_h}{V} = 1$$

The ratio of volume of each component to the total volume of the infinitesimal observation window is defined at the volume fraction of the component $\phi_i(\boldsymbol{x},t)$, for $i = n, e, h$, and determines the content of nuclear constituents – nucleoplasm, euchromatin and heterochromatin at each point $\boldsymbol{x}$ and time t in the nucleus. Thus,

$$\phi_n + \phi_e + \phi_h = 1$$

Thus, the physical state of the nucleus at any point can be defined by volume fractions of any two nuclear constituents, with the third constrained via the above equation. Equivalently, the physical state of the nucleus at any point can be completely defined via two independent variables – (i) volume fraction of nucleoplasm $\phi_n(\boldsymbol{x},t)$, and (ii) difference between the volume fractions of heterochromatin and euchromatin $\phi_d(\boldsymbol{x},t) = \phi_h(\boldsymbol{x},t) - \phi_e(\boldsymbol{x},t)$. ϕ_d can be considered an order parameter which when negative implies a euchromatin rich phase and when positive implies a more condensed heterochromatin rich phase. While the change of the variables is entirely equivalent mathematically, physiologically such a description permits a natural definition of the movement of the two mobile species in the nucleus – nucleoplasm or water, and the epigenetic marks of acetylation or methylation.

S1.2 Free energy landscape of the nucleus:

In terms of the independent variables $\phi_d(\boldsymbol{x},t)$ and $\phi_n(\boldsymbol{x},t)$, the free energy density at any point $\boldsymbol{x}$ can be expressed as $W(\phi_n, \phi_d, \nabla\phi_n, \nabla\phi_d)$ where we have also incorporated the energetic considerations associated with the phase interfaces via the spatial gradients of the volume fractions. Specific form of the free energy can be invoked by considering the various energetic contributions in the nucleus such as,

$$W = \underbrace{W_{CCI}(\phi_d, \phi_n)}_{\text{chromatin-chromatin interactions}} - \underbrace{V_L \phi_h e^{-\frac{d}{d_0}}}_{\text{chromatin-lamina interactions}} + \underbrace{\frac{\eta_n}{2}|\nabla\phi_n|^2 + \frac{\eta_d}{2}|\nabla\phi_d|^2}_{\text{Interfacial energy}} \qquad \text{(S1)}$$

- The first term in Eq S1 arises from the competition between entropy and enthalpy of mixing heterochromatin and euchromatin phases. It is equivalent to the Flory-Huggins free energy description, as discussed in subsection S1.3. This term gives rise to the double-well form of the free energy landscape, as shown in the contour plot in Figure 1b. The two wells, shown as red and blue dots, are the energy minima corresponding to the two stable phases of

chromatin - a water-rich, loosely packed euchromatin phase ($\phi_h = 0$) or a compacted water-devoid heterochromatin phase ($\phi_h = \phi_h^{max}, \phi_n \sim 0$). Here ϕ_h^{max} denotes the extent of compaction in the heterochromatin phase. Any initial chromatin configuration will spontaneously phase separate into heterochromatin and euchromatin domains (red arrows).

- The second term captures the interactions between the chromatin and the lamina via chromatin anchoring proteins (HDAC3, LAP2β, emerin, etc. [1-3]) with parameter V_L denoting the strength of these anchoring interactions. Since these interactions are mediated by proteins anchored on the lamina, we take an interaction strength that is most robust at the nuclear periphery (distance from lamina $d = 0$) and decays exponentially away from the lamina over a length scale d_0 (schematically shown in Figure S1). Note that the exact decay characteristics do not really affect the model qualitatively. We choose exponential form as a generalized minimal-parameter decay. The negative sign permits an energetic attraction for the heterochromatin phase along the nuclear periphery. We chose the heterochromatin phase specifically to interact with the lamina since the chromatin domains preferentially associating with the nuclear lamina are linked to transcriptional repression and an increased histone methylation [2, 4-6].

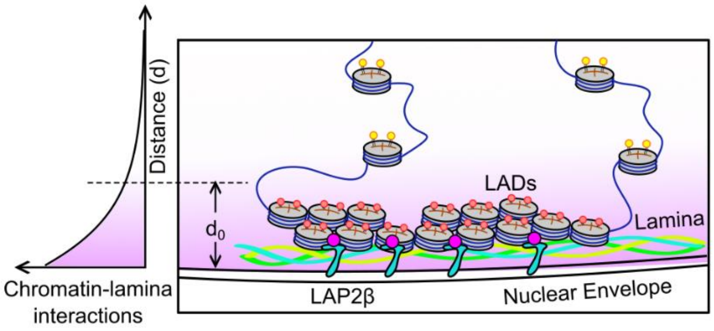

Figure S1: Schematic representation of the energetic interactions between chromatin and the lamina mediated by anchoring proteins like LAP2β.

- The last term in Eq S1 denotes the energy penalty associated with forming phase boundaries between the euchromatin and heterochromatin phases as they separate. The term $\eta_{n,d}$ is the increase in the energy due to formation of a unit width of the interface. Note that the term $|\nabla\phi_{n,d}|$ is the magnitude of the slope of the interface. Notably, as $\eta_{n,d}$ increases, there is a greater penalty on formation of sharp interfaces, resulting in more smooth interfaces which are wider. Thus η_n and η_d directly control the width and the energy of the phase boundaries. In our simulations, we choose $\eta_n = \eta_d = \eta$.

The total free energy of the nucleus of volume Ω can be written, after incorporating the work done in exchanging water between the nucleus and the cytoplasm, as,

$$\Pi[\phi_n, \phi_d] = \int_\Omega W(\phi_n, \phi_d, \nabla\phi_n, \nabla\phi_d) dV - \int_{\partial\Omega_p} \bar{\mu}_n I^n dA \qquad (S2)$$

Here, $\partial\Omega_p$ denotes the surface of the nucleus where pores and channels allow exchange of water between nucleus and cytoplasm by maintaining an ‘external’ chemical potential $\bar{\mu}_n$. I^n is the volume of water entering per unit surface area into the nucleus, as a result. The double-well energy landscape described by Eq 1 and Figure 1b, drive the time evolution (red arrows) of chromatin from an initial configuration (say corresponding to the red dot in Figure 1b) into the two energy minimal wells corresponding to the two chromatin phases. The gradients of the free energy in the ϕ_n-ϕ_d variable space (shown by the contour plot in Figure 1b) provide the driving force for the time-evolution of chromatin organization towards the steady-state. This driving force is called the chemical potential and is written using variational principles as,

$$\mu_n(\boldsymbol{x},t) = \frac{\delta W}{\delta \phi_n} = \frac{\partial W}{\partial \phi_n} - \boldsymbol{\nabla} \cdot \left(\frac{\partial W}{\partial \nabla \phi_n}\right)$$
$$\mu_d(\boldsymbol{x},t) = \frac{\delta W}{\delta \phi_d} = \frac{\partial W}{\partial \phi_d} - \boldsymbol{\nabla} \cdot \left(\frac{\partial W}{\partial \nabla \phi_d}\right) \qquad (S3)$$

where, μ_n is the chemical potentials of nucleoplasm driving its kinetics as described in Section S1.4. And μ_d is the chemical potential for the order parameter ϕ_d evolving the epigenetic marks in a conserved manner as discussed in the sub-section S1.5.

S1.3 Specific form of the energetic contribution from chromatin-chromatin interactions

Before discussing the role of chemical potentials μ_d and μ_n in spatiotemporal evolution of chromatin organization, we describe the specific form of energetic contributions $W_{CCI}(\phi_d, \phi_n)$ arising from the chromatin-chromatin interactions in Eq S1. The chosen form of energetic contribution must account for a ternary mixture of the three nuclear constituents, with volume fractions - ϕ_e, ϕ_h and ϕ_n. An energy landscape can suitably be constructed to obtain two coexisting phases:

(i) Water rich euchromatin phase with $\phi_n > \phi_e, \phi_h$, and $\phi_h \to 0$.
(ii) Water poor heterochromatin phase with $\phi_h > \phi_e$,ϕ_n and $\phi_h \to \phi_h^{max}$. Also, ϕ_n is very small.

The Flory Huggins model for the energetics of a mixture of polymer in a solvent can be used as a description of the energy landscape. It incorporates the competition between the change in entropy due to the mixing process and the change in enthalpy due to interactions such as bond formation between the polymer and the solvent. The change in free energy density of a ternary mixture separating into two phases, accounting for entropic and enthalpic contributions is given as [7, 8],

$$W_{CCI}(\phi_d, \phi_n) = \frac{k_B T}{\Omega}\left[\phi_e^2 + \underbrace{\frac{\phi_h}{N}\ln\phi_h + \phi_n \ln(\phi_n)}_{\text{Entropic Contribution}} + \underbrace{\chi\phi_h\phi_n}_{\text{Enthalpic Contribution}}\right] \qquad (S4)$$

where, N is the degree of polymerization. For a particularly large polymer such as chromatin in the nucleoplasm, $N \to \infty$ thereby lowering the entropic contribution to the free energy. On a (ϕ_d, ϕ_n) phase space, the contour plot of the energy density $W(\phi_d, \phi_n)$ is shown in Figure S2, left. Also note that, k_B is the Boltzmann constant, T is the temperature and Ω is the volume of individual chromatin particles. The coefficient $k_B T/\Omega$ acts as an energy scaling factor.

The Flory-Huggins form of free energy is appropriate when considering only the enthalpic and entropic contributions to the free energy. *It has been shown that locally driven energy consuming/producing activity, such as motor activity or transcription, or ATP consumption, can drive an 'activity-induced' segregation of the chromatin phases [9]. To account for such, more general, mechanisms of phase-separation it becomes more suitable to adopt a generalized simplified biquadratic form of energy landscape. Several polynomial descriptions of double-well energy functions emulating the Flory-Huggins form have been proposed and utilized previously [10-13].* To capture the biphasic (hetero- and euchromatin) phase-separation of a ternary mixture (ϕ_e, ϕ_h, ϕ_n) we adopt a simplified biquadratic double-well energy as,

$$W_{CCI}(\phi_d, \phi_n) = \frac{k_B T}{\Omega}\left[\phi_e^2 + {\phi_h}^2(\phi_h^{max} - \phi_h)^2\right] \qquad (S5)$$

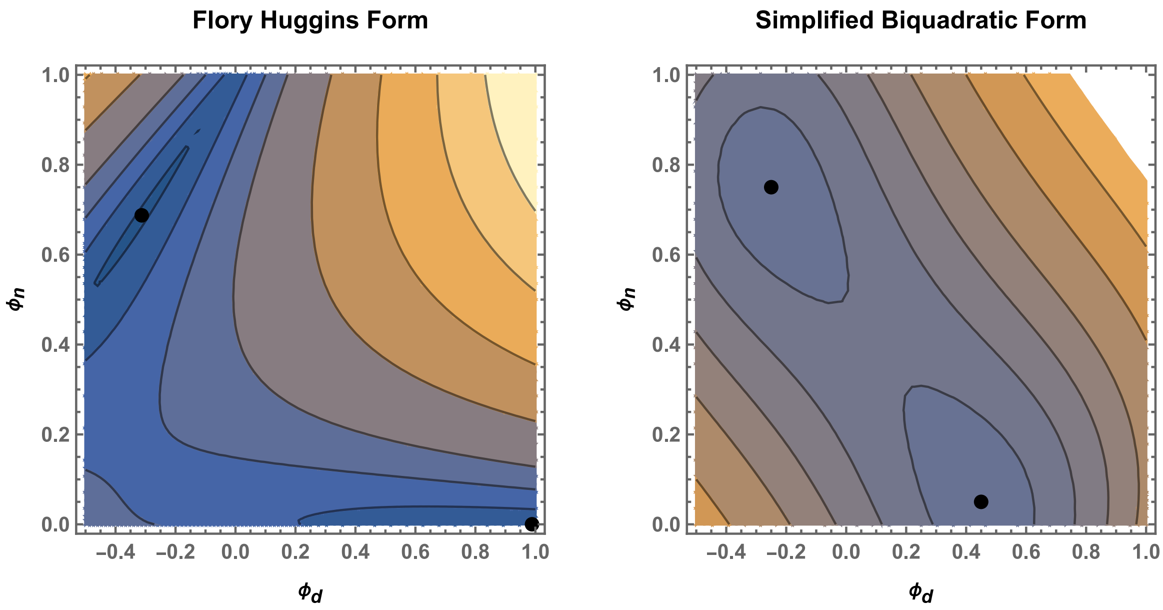

Figure S2: The energy landscape shown as a contour plot on a (ϕ_d, ϕ_n) phase space. (a) Energy landscape considering Flory Huggins description given by Eq S4, and (b) simplified biquadratic double well description given by Eq S5.

The contour plot of the adopted free energy form (Eq S5) on a (ϕ_d, ϕ_n) phase space (Figure S2, right), shows a similar location of two energy wells as the Flory-Huggins description. However, the simplification additionally allows (a) easier numerical implementation as the log terms may become undefined due to numerical errors, and (b) a better control on the location of wells, since the well locations are directly defined by the value of the parameter ϕ_h^{max}. Due to these advantages, we use the free energy description given by Eq S5.

S1.4 Diffusion and reaction kinetics

The dynamic evolution of the nucleus towards the steady-state is governed in a time-dependent fashion by a combination of diffusion and reaction kinetics (Figure 1b, bottom panel). The local conservation of reactively inert nucleoplasm content relates the time evolution of local nucleoplasm volume fraction with its nominal volumetric flux $\mathcal{J}^n$ as, $\dot{\phi}_n = -\nabla . \mathcal{J}^n$. Fick's first law gives the volumetric flux of nucleoplasm as $\mathcal{J}^n = -M_n \nabla \mu_n$ in terms of the gradient of the chemical potential of nucleoplasm where M_n denotes the mobility of nucleoplasm in the nucleus, which is related to the dissipation that occurs when water flows through the porous nuclear microstructure. Thus,

$$\frac{\partial \phi_n}{\partial t} = \underbrace{M_n \nabla^2 \mu_n}_{\text{diffusion}} \qquad (S6)$$

The epigenetic reaction kinetics controls the methylation or acetylation levels of the histone tail marks. Heterochromatin, which is rich in methylation marks on the histone tails, can be converted into euchromatin phase, where histones are marked by an increased acetylation level. This process encompasses the removal of methylation marks on the histone tails called demethylation via proteins classified as histone demethylase (HDM), followed by acetylation of the histone tails, via histone acetyltransferase (HAT) as shown in Figure S3. We classify both these processes together as the acetylation of the histone – occurring at a rate Γ_{ac} – which converts

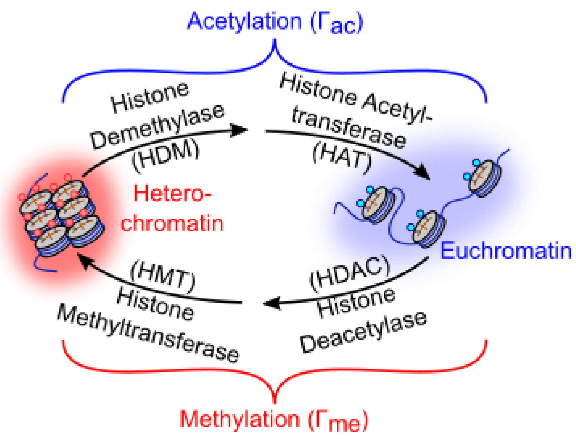

Figure S3: Epigenetic factors catalyze the reactions leading to interconversion of euchromatin and heterochromatin. The reactions are broadly methylation of euchromatin and acetylation of heterochromatin.

heterochromatin into euchromatin. Conversely euchromatin is converted into heterochromatin by first the deacetylation (via histone deacetylase, HDAC) followed by methylation (via histone methyltransferase, HMT) at a cumulative rate Γ_{me} as shown in Figure S3. Distinct from the conservative evolution in Eq S7, the interconversion of chromatin phases via epigenetic reactions changes the relative content of heterochromatin and euchromatin such that,

$$\left.\frac{\partial \phi_d}{\partial t}\right|_{\text{epigen}} = 2(\Gamma_{me}\phi_e - \Gamma_{ac}\phi_h) \qquad (S7)$$

In the reaction kinetics via Eq S7, we have considered that any heterochromatin-euchromatin interconversion occurs at a constant rate. However, this does not account for the chromatin-chromatin interaction energetics due to which heterochromatin-heterochromatin or euchromatin-euchromatin neighbors are more stable over euchromatin-heterochromatin neighbors. Therefore, if acetylation results in more euchromatin-euchromatin neighbors it will be more favorable than if it results in unlike-marked neighbors. Therefore, the acetylation and methylation reactions are additionally influenced by the specific location of the reaction site within the genome. The role of energetics in driving the reaction kinetics is discussed in more detail with specific schematic example in Section S1.6. Using qualitative examples given in Section S1.6, and with precise theoretical derivation in Section S1.7, we realize that such neighborhood dependent reaction kinetics effectively emulate a diffusion-like evolution of epigenetic marks. In other words, chromatin evolves in time as if the epigenetic marks of acetylation and methylation are diffusing spatially dependent on the gradients of their chemical potential μ_d. Such evolution is written as,

$$\left.\frac{\partial \phi_d}{\partial t}\right|_{\text{cons}} = M_d \nabla^2 \mu_d \qquad (S8)$$

Here, M_d is the mobility of epigenetic marks in the nucleus. Note that mobility M is related to the diffusivity as $D = \frac{M k_B T}{\Omega}$.

Lastly, transcription by RNAPII activity requiring ATP-based energy expenditure, is known to supercoil chromatin fiber thereby resulting in active extrusion of DNA through cohesin rings. Since extruded chromatin loops are transcriptionally active, such extrusion within the euchromatin phase does not alter the gene expression. However, extrusion of transcriptionally silent heterochromatin into chromatin loops switches the transcriptional state of chromatin pulling out the silenced genes near the heterochromatin-euchromatin boundary into the euchromatin phase (Figure 1c). Chromatin extrusion at the phase boundaries happens in two steps (Figure 1c, 6a):

1. Cohesin rings entrap a portion of DNA fiber along the interface due to a balance between its loading (with reaction rate Γ_l) via NIPBL/MAU2 and unloading (with reaction rate Γ_{ul}) via WAPL/PDS5. The overall rate of cohesin loading can be written as $\Gamma_{coh} = \Gamma_l - \Gamma_{ul}$.

2. This is followed by the active extrusion of supercoiled loops of chromatin through the cohesin rings via the transcription due to RNAPII, at a rate denoted by Γ_{tr}.

Altogether, $\Gamma_a = \Gamma_{tr} \times \Gamma_{coh}$ denotes the overall the rate at which the chromatin extrusion converts heterochromatin into euchromatin via the two-step process (Figure 1c). Thus, we can write the transcriptionally-dependent conversion of the chromatin phases as,

$$\left.\frac{\partial \phi_d}{\partial t}\right|_{\text{transcription}} = -2\left(\Gamma_a e^{-\left(\frac{\phi_h - \frac{\phi_h^{\max}}{2}}{2\,\Delta\phi}\right)^2} \phi_h\right) \qquad (S9)$$

Where, the exponential factor ensures that the transcription-based chromatin extrusion is spatially restricted to a narrow region where the volume fraction of heterochromatin is ${\phi_h^{\max}}/{2} - \Delta\phi \le \phi_h \le {\phi_h^{\max}}/{2} + \Delta\phi$, while peaking at the interface ($\phi_h = \phi_h^{\max}/2$). Combining Eq S7-S9, the time-evolution of the order parameter ϕ_d can be written as,

$$\frac{\partial \phi_d}{\partial t} = \underbrace{M_d \nabla^2 \mu_d}_{\text{diffusion}} + 2\left(\underbrace{\Gamma_{me}\phi_e - \Gamma_{ac}\phi_h}_{\substack{\text{epigenetic}\\ \text{regulation}}} - \underbrace{\Gamma_a e^{-\left(\frac{\phi_h - \frac{\phi_h^{\max}}{2}}{2\,\Delta\phi}\right)^2} \phi_h}_{\substack{\text{active chromatin}\\ \text{extrusion}}}\right) \qquad (S10)$$

Equations S3, S6 and S10 together form the time-dependent mathematical set of governing equations describing the spatiotemporal evolution of chromatin organization in the nucleus. As a ready reference, we have here listed the mathematical symbols used in our model, their physical meaning, and units in Table S1.

Table S1: List of mathematical symbols

	Symbol	Physical Interpretation	SI Unit	Remarks
	x	Spatial variable, two-dimensional vector	m	
	t	Time variable	s	
Volume Fractions	ϕ_h	Volume fraction of heterochromatin	1	Defined in Section S1.1
	ϕ_e	Volume fraction of euchromatin	1	
	ϕ_n	Volume fraction of nucleoplasm	1	
	ϕ_d	$= \phi_h - \phi_e$, difference between heterochromatin and euchromatin volume fractions, order parameter	1	
Energetic contributions	W	Total free energy density of chromatin organization	J/m^3	Defined via Eq S1
	W_{CCI}	Free energy density due to chromatin-chromatin interactions	J/m^3	Defined via Eq S5
	V_L	Strength of chromatin-lamina interactions	J/m^3	Defined in Section S1.2
	d	Distance of a point from the nuclear periphery	m	
	d_0	Length-scale of chromatin-lamina interactions – the distance over which effect of chromatin-anchoring proteins vanishes	m	
	η	Energy penalty on formation of sharp interface	J/m	
Diffusion kinetics	$\mu_{n,d}$	Chemical potential $= \delta W/\delta\phi_{n,d}$ – driving force for energy-reducing passive diffusion kinetics	J/m^3	Defined via Eq S3
	$\mathcal{J}^{n,d}$	Nominal volumetric flux of mobile species – defined as volume of species flowing per unit local area per unit time	m^3/m^2s	Defined in Section S1.4

	$M_{n,d}$	Mobility of nucleoplasm or epigenetic marks in the nucleus	$\frac{m^2/s}{J/m^3}$	
	$D_{n,d}$	Diffusivity of nucleoplasm of epigenetic marks in the nucleus	m^2/s	$= Mk_BT/\Omega$
Reaction kinetics	Γ_{me}	Reaction rate of methylation, defined as deacetylation + methytransferase activity	s^{-1}	Defined in Section S1.4
	Γ_{ac}	Reaction rate of acetylation, defined as demethylation + acetyltransferase activity	s^{-1}	
	Γ_a	Rate of chromatin extrusion driven by transcription-mediated supercoiling	s^{-1}	

S1.5 Rescaling the governing equations

The governing equations derived in the previous sections reveal intrinsic length and time scales within the model, which we next use to obtain the rescaled, non-dimensional set of governing equations.

The reaction-diffusion kinetics from Eq S10 results in a characteristic length scale determined by the reaction-diffusion kinetics i.e $\ell_{RD} = \sqrt{D/\Gamma_{ac}} = \sqrt{Mk_BT/\Omega\Gamma_{ac}}$. Interestingly, it can also be seen that another intrinsic length scale emerges from the competition between the interfacial and bulk mixing energies from Eq S1, i.e. the width of the interface $\ell_{int} = \sqrt{\eta\Omega/k_BT}$. In our simulations, and from the theory discussed later in Section S4, we find that the reaction-diffusion length plays a significant role in determining the heterochromatin domain sizes and their spacing. Therefore, we choose to rescale all lengths with respect to ℓ_{RD}, such that $\tilde{\boldsymbol{x}} = \boldsymbol{x}/\ell_{RD}$. Further, the reaction rates offer an intrinsic time scale for the system of equations, such that all times are rescaled as $\tilde{t} = t\Gamma_{ac}$. Lastly, k_BT/Ω which is the coefficient of energy of chromatin phase interactions in Eq S5 provides the energy scaling such that all energy densities are written as $\widetilde{W} = W\Omega/k_BT$.

Rescaling Eq S1,

$$\widetilde{W} = \underbrace{\left[\phi_e^2 + \phi_h^2(\phi_h^{\max} - \phi_h)^2\right]}_{\text{chromatin-chromatin interactions}} - \underbrace{\tilde{V}_L\phi_h e^{-\frac{d}{d_0}}}_{\text{chromatin-lamina interactions}} + \underbrace{\frac{1}{2}\frac{\eta\Omega}{k_BT}\frac{\Omega\Gamma_{ac}}{Mk_BT}|\nabla\phi_n|^2 + \frac{1}{2}\frac{\eta\Omega}{k_BT}\frac{\Omega\Gamma_{ac}}{Mk_BT}|\nabla\phi_d|^2}_{\text{Interfacial energy}}$$

Here, $\tilde{V}_L = V_L\Omega/k_BT$ is the rescaled strength of chromatin-lamina anchoring interactions. Note that the coefficient of the interfacial energy terms can be rewritten in terms of the ratio of the length scales $\frac{\ell_{int}}{\ell_{RD}} = \delta$. Physically, the parameter δ is a rescaled measure of the width of the interface. Thus,

$$\widetilde{W} = \underbrace{\left[\phi_e^2 + \phi_h^2(\phi_h^{\max} - \phi_h)^2\right]}_{\text{chromatin-chromatin interactions}} - \underbrace{\tilde{V}_L\phi_h e^{-\frac{d}{d_0}}}_{\text{chromatin-lamina interactions}} + \underbrace{\frac{\delta^2}{2}|\nabla\phi_n|^2 + \frac{\delta^2}{2}|\nabla\phi_d|^2}_{\text{Interfacial energy}} \qquad (S11)$$

The rescaled chemical potentials from Eq S3 are,

$$\tilde{\mu}_n(\tilde{\boldsymbol{x}},\tilde{t}) = \frac{\partial\widetilde{W}}{\partial\phi_n} - \boldsymbol{\nabla}\cdot\left(\frac{\partial\widetilde{W}}{\partial\nabla\phi_n}\right)$$

$$\tilde{\mu}_d(\tilde{\boldsymbol{x}},\tilde{t}) = \frac{\partial\widetilde{W}}{\partial\phi_d} - \boldsymbol{\nabla}\cdot\left(\frac{\partial\widetilde{W}}{\partial\nabla\phi_d}\right)$$

Expanding these,

$$\tilde{\mu}_n(\tilde{\boldsymbol{x}},\tilde{t}) = -\phi_e - \phi_h(\phi_h^{max} - \phi_h)(\phi_h^{max} - 2\phi_h) - \frac{1}{2}\tilde{V}_L e^{-\frac{d}{d_0}} - \delta^2\nabla^2\phi_n$$
$$\tilde{\mu}_d(\tilde{\boldsymbol{x}},\tilde{t}) = -\phi_e + \phi_h(\phi_h^{max} - \phi_h)(\phi_h^{max} - 2\phi_h) - \frac{1}{2}\tilde{V}_L e^{-\frac{d}{d_0}} - \delta^2\nabla^2\phi_d \quad (S12)$$

Lastly, we rescale the kinetics equations such that,

$$\frac{\partial\phi_n}{\partial\tilde{t}} = \underbrace{\nabla^2\tilde{\mu}_n}_{\text{diffusion}}$$
$$\frac{\partial\phi_d}{\partial\tilde{t}} = \underbrace{\nabla^2\tilde{\mu}_d}_{\text{diffusion}} + 2\left(\underbrace{\tilde{\Gamma}_{me}\phi_e - \phi_h}_{\substack{\text{epigenetic}\\ \text{regulation}}} - \underbrace{\tilde{\Gamma}_a e^{-\left(\frac{\phi_h - \frac{\phi_h^{max}}{2}}{2\,\Delta\phi}\right)^2}\phi_h}_{\substack{\text{active chromatin}\\ \text{extrusion}}}\right) \quad (S13)$$

Note that in the second part of Eq S13, all reaction rates have also been rescaled with respect to the time scale such that $\tilde{\Gamma}_{me} = \frac{\Gamma_{me}}{\Gamma_{ac}}$ and $\tilde{\Gamma}_a = \frac{\Gamma_a}{\Gamma_{ac}}$. To solve the rescaled set of governing equations, Eq S12 and Eq S13 are solved together subjected to boundary conditions of no flux of nucleoplasm or epigenetic marks across all boundaries. For the purposes of numerical implementation, these equations are converted into weak-form suitable for a suitable for finite-element solver implementation. We then used COMSOL Multiphysics with a 'Weak Form PDE' module for the solution of the equations. The boundary conditions, and the list of non-dimensional parameters used in our model are discussed in detail in Section S8.

S1.6 <u>Polymer analogy of the roles played by reaction and diffusion kinetics</u>

As discussed in the previous section, we have incorporated the kinetics of both diffusive and reactive nature – the former being conservative, i.e. it does not change the net amount of heterochromatin and euchromatin in the nucleus, while the latter non-conservative since it allows interconversion of the two phases (Figure S3). Here we explain in detail the kinetics underlying the non-conservative and conservative dynamics of nucleosomes in the chromatin polymer.

Firstly, we consider the diffusion of nucleoplasm via the conservative kinetics given by Eq S6. If there is no flux of water occurring across the nuclear lamina, the net amount of nucleoplasm in the nucleus $\bar{\phi}_n$ remains a constant. Locally the effect of nucleoplasm movement allows methylated histones to come together resulting in their compaction (Figure S4a). Thus, the conservative diffusion of nucleoplasm allows coarsening of heterochromatin domains keeping the euchromatin phase water rich.

Before discussing the effective diffusion of epigenetic marks via the conservative kinetics (Eq S7), we focus on the epigenetic reaction kinetics described via Eq S8. The role of epigenetic reaction kinetics is non-conservative since it allows an interconversion of euchromatin to heterochromatin via a reaction rate Γ_{me} and heterochromatin to euchromatin via a reaction rate Γ_{ac} (Figure S4b). Note that these interconversions require multiple steps, such as demethylation followed by acetylation, or deacetylation followed by methyltransferase activity (Figure S3). While these reactions do not affect the total amount of water or DNA in the nucleus, they change the individual amounts of heterochromatin and euchromatin. For instance, increase in Γ_{ac} (Γ_{me}) will increase the

overall euchromatin (heterochromatin) in the nucleus, without changing the total amount of chromatin.

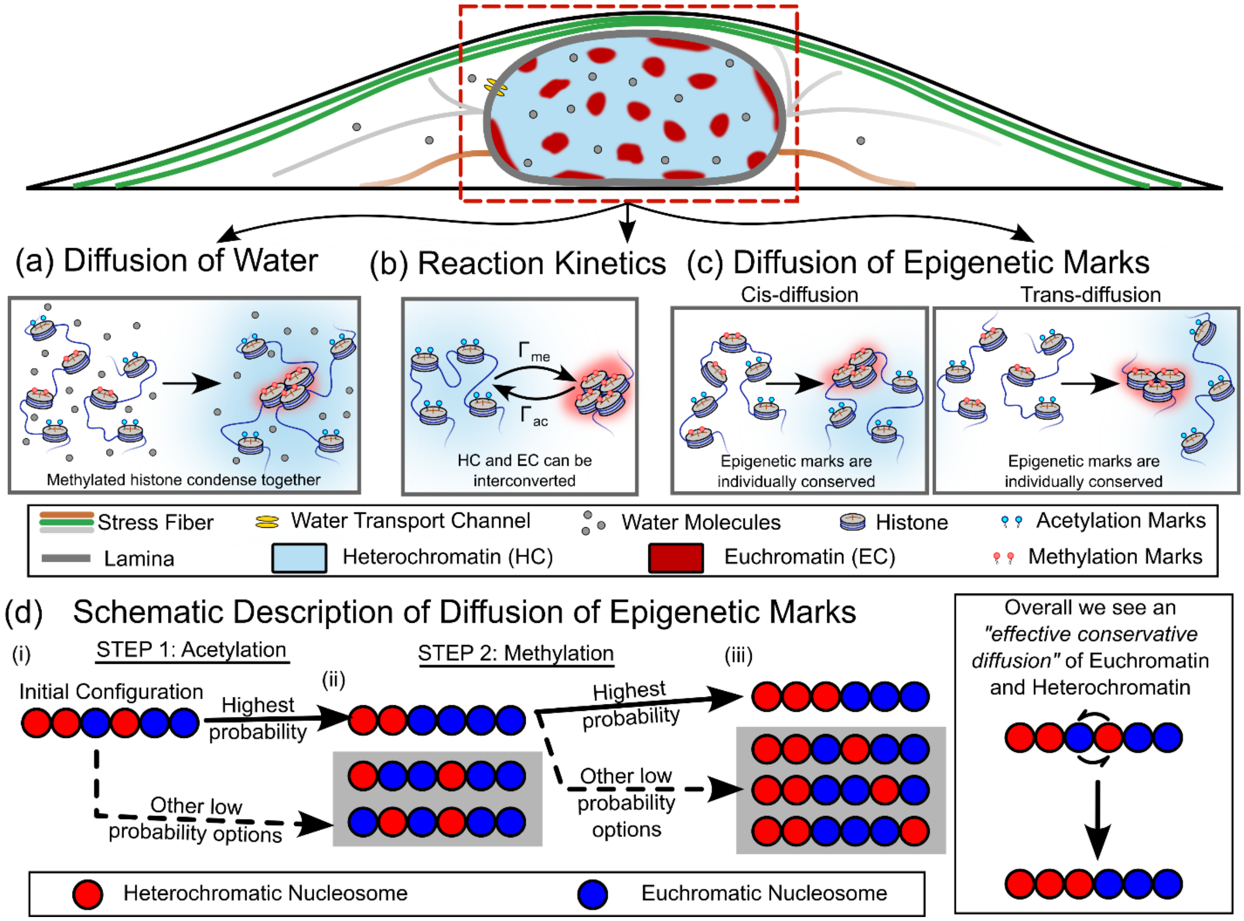

Figure S4: A schematic depicting the individual roles of the diffusion and reactions kinetics incorporated into the heterochromatin organization model. (a) Conservative diffusion of water which can redistribute molecules of water within the nucleus without changing the total amount of water, or total amount of hetero- or euchromatin in the nucleus. (b) Non-conservative reaction kinetics of histone acetylation and methylation, which allows an interconversion of chromatin phases. This changes the individual amounts of heterochromatin and euchromatin in the nucleus without changing the total amount of DNA. The reaction rates determine the ratio of heterochromatin to euchromatin at steady state. (c) The chromatin-chromatin interactions contribute to the reaction kinetics effectively driving a conservative evolution of epigenetic marks, which we call 'diffusion of epigenetic marks'. (d) The diffusion of epigenetic marks is an overall result of reaction kinetics coupled with preference of like-like neighbor over unlike ones, thereby effectively rendering reactions ate certain sites more probable than others.

The presence of epigenetic reactions has an additional energetics influenced component. When considering the reaction kinetics due to epigenetic regulation we have to account for the fact that the nucleosomes that are in close proximity to each other can interact. These interactions might stem from histone-bridging proteins such as HP1 [14, 15], binding complexes such as SAGA [16], or even ionic interactions due to the presence of post-translational modifications. The interactions are captured in our model as chromatin-chromatin interactions and give rise to the energy landscape described in Figure 1b. Because of chromatin-chromatin interaction energetics, heterochromatin-heterochromatin or euchromatin-euchromatin neighbors are more stable than euchromatin-heterochromatin neighbors. Thus, the acetylation and methylation reactions effectively depend on the specific location of the nucleosome within the genome.

The role of energetics can be better understood via the schematic shown in Figure S4d. Say a portion of chromatin polymer at some point in time looks like the initial configuration shown in Figure S4d(i) (in the figure, heterochromatin is shown as red and euchromatin blue). Without loss of generality, let us say that the first step is conversion of heterochromatin into euchromatin (acetylation). There are various heterochromatin sites which can be converted into euchromatin (shown in the grey box), but the configuration shown in Figure S4d (ii) has the highest probability of occurring. This is because it maximizes like-nucleosome neighbors and thus reduces the energy of chromatin-chromatin interactions the most. The next step could either involve converting heterochromatin to euchromatin or vice versa. If a heterochromatin to euchromatin flip occurs, in our meso-scale model its effect is captured by the non-conservative reaction (Eq S8). On the other hand, if a euchromatin to heterochromatin flipping occurs, the possible configurations attainable are shown in the grey box. The configuration with highest probability of occurrence is if all heterochromatic nucleosomes are segregated from the euchromatic ones, as shown in Figure S4d (iii). Thus, over the course of these two steps, we see that an effective conservative rearrangement of heterochromatin and euchromatic nucleosomes can occur which leads to coarsening of the two phases. This conservative evolution effectively is captured in our model as 'diffusion of epigenetic marks'.

Note that the sequence of events described here gives rise to effective diffusion of epigenetic marks along the chain of polymer – labelled cis-diffusion in Figure S4c. A similar reaction kinetics driven epigenetic diffusion can occur between nucleosomes that are close in the 3D space, although not neighbors on the chromatin polymer chain. Such an event, as shown in Figure S4c, can be called trans-diffusion, and can similarly occur.

To support this qualitative description of events, in the next section we show how the presence of reaction rates dependent on the energetic interactions of nucleosomes can give rise to effective 'diffusion of epigenetic marks'.

S1.7 <u>Neighborhood dependent reaction-kinetics emulates the 'diffusion of epigenetic marks'</u>

In this subsection, we will theoretically prove that when the epigenetic reaction kinetics becomes neighborhood dependent, as discussed in subsection S1.6, we recover the spatiotemporal evolution described by Eq S10, used in our continuum chromatin dynamics model. To do so, first we will explain the stochastic events describing the conservative diffusive and non-conservative reaction kinetics.

<u>Simple diffusion</u>: In a 1D setting (to simulate a polymer), consider the series of empty sites that a randomly walking particle can occupy, as shown in Figure S4.1. Let us focus on a site at location x with the left and right neighbors located at $x - dx$ and $x + dx$. Let $P(x)$ be the probability that the site at location x is occupied. For brevity, the probability that the left site is occupied, $P(x - dx)$ is denoted as P^{-}, while the probability that the right site is occupied $P(x - dx)$ is denoted as P^{+}.

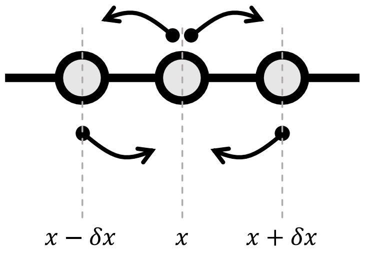

Figure S4.1: Random walking particle emulates diffusion.

There are four possible events that can occur (Figure S4.1) – if the site at position x is occupied, the particle can either move to the left or right given that those sites are empty, or if the site at position x is free particle from left or right neighboring site can

move in, provided that those sites are occupied. Then the probability of particle occupying site x evolves in time δt as,

$$\frac{\partial P(x,t)}{\partial t} = \frac{1}{\delta \mathrm{t}}\left[-\frac{1}{2}P(1-P^-) - \frac{1}{2}P(1-P^+) + \frac{1}{2}P^-(1-P) + \frac{1}{2}P^+(1-P)\right]$$

Expanding the left and right probabilities via Taylor expansion to the third order,

$$\frac{\partial P(x,t)}{\partial t} = \frac{1}{\delta \mathrm{t}}\left[-P(x) + \frac{1}{2}\left\{P(x) + \frac{dP}{dx}\delta x + \frac{d^2P}{dx^2}\frac{(\delta x)^2}{2} + P(x) - \frac{dP}{dx}\delta x + \frac{d^2P}{dx^2}\frac{(\delta x)^2}{2}\right\}\right]$$

$$\frac{\partial P(x,t)}{\partial t} = \underbrace{\frac{(\delta x)^2}{2\delta t}}_{\text{Diffusivity}} \frac{d^2P}{dx^2} \tag{S14}$$

Thus, the spatiotemporal evolution of randomly walking particles follows the diffusive kinetics.

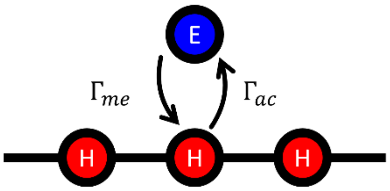

Figure S4.2: Reaction Kinetics.

Reaction kinetics: In a 1D setting, now consider that the nucleosomes can be of two flavors: heterochromatic (H) and euchromatic (E). P_H is the probability that the nucleosome is heterochromatic. The probability that nucleosome is euchromatic is $P_E = 1 - P_H$. The two nucleosome species can be interconverted at a rate Γ_{ac} or Γ_{me} (dimension of s^{-1}). Note that nucleosomes are not moving in space, only their flavor changes in time. For now, let us assume that these conversion rates are constant. Then the probability that nucleosome at site x is heterochromatic $P_H(x,t)$ evolves in time as,

$$\frac{\partial P_H(x,t)}{\partial t} = -\Gamma_{ac}P_H + \Gamma_{me}P_E \tag{S15}$$

It should be noted that Eq S15 is analogous to the non-conservative reaction kinetics in our continuum model as given by Eq S7. At steady state $\left(\frac{\partial P_H}{\partial t} = 0\right)$, the probability of seeing a heterochromatic nucleosome at a site is $P_H = \frac{\Gamma_{me}}{\Gamma_{ac}+\Gamma_{me}}$. This is again analogous to the theoretical average heterochromatin content in the nucleus, derived from our model (see Section S3).

Reaction kinetics with neighborhood dependent energetics: Now let us consider the case where the rate of acetylation and methylation are not constant, but dependent on whether the neighbors are similar or dissimilar. This can happen because the conversion requires breaking bonds between neighbors. Say if the nucleosome is H (heterochromatic), and needs to convert to E (euchromatic), there are four possible options:

	Configuration		Change in energy	Acetylation rate
1	HHH	Need to break two hetero-hetero bonds	$\Delta \mathrm{E} = 2\mathrm{E}_{HH}$	Γ_{ac}
2	HHE	Need to break one hetero-hetero and one eu-hetero bond	$\Delta \mathrm{E} = \mathrm{E}_{HH} + E_{EH}$	$\sim \Gamma_{ac} + \Delta\Gamma$
3	EHH			

4	EHE	Need to break two eu-hetero bonds	$\Delta E = 2E_{EH}$	$\sim \Gamma_{ac} + 2\Delta\Gamma$

Since like-marked nucleosomes interact strongly as compared to unlike marked nucleosomes, $E_{EH} < E_{HH}, E_{EE}$. Thus, acetylation rate is higher $(\Delta\Gamma > 0)$ when a bond between unlike nucleosomes is broken than if that between like-marked nucleosome is broken. Similarly, we can have four possible options for Γ_{me}. Considering these eight possible options after taking into account the effects of the neighbors, the probability that nucleosome at site x is heterochromatic evolves in time as,

$$\frac{\partial P_H(x,t)}{\partial t} = -P_H\left[\underbrace{P_H^+P_H^-\Gamma_{ac}}_{HHH} + \underbrace{P_H^+P_E^-(\Gamma_{ac}+\Delta\Gamma)}_{HHE} + \underbrace{P_E^+P_H^-(\Gamma_{ac}+\Delta\Gamma)}_{EHH} + \underbrace{P_E^+P_E^-(\Gamma_{ac}+2\Delta\Gamma)}_{EHE}\right] + P_E\left[\underbrace{P_E^+P_E^-\Gamma_{me}}_{EEE} + \underbrace{P_E^+P_H^-(\Gamma_{me}+\Delta\Gamma)}_{EEH} + \underbrace{P_H^+P_E^-(\Gamma_{me}+\Delta\Gamma)}_{HEE} + \underbrace{P_H^+P_H^-(\Gamma_{me}+2\Delta\Gamma)}_{HEH}\right] \quad \text{(S16)}$$

Here, $P_{H(E)}^{+(-)}$ is the probability that the right (left) neighbor is heterochromatic (euchromatic). For simplification, we begin with the first bracket term on the right side of Eq S16:

$$-P_H[P_H^+P_H^-\Gamma_{ac} + P_H^+P_E^-(\Gamma_{ac}+\Delta\Gamma) + P_E^+P_H^-(\Gamma_{ac}+\Delta\Gamma) + P_E^+P_E^-(\Gamma_{ac}+2\Delta\Gamma)]$$

$$= -P_H[\Gamma_{ac}\{P_H^+P_H^- + P_H^+P_E^- + P_E^+P_H^- + P_E^+P_E^-\} + \Delta\Gamma\{P_H^+P_E^- + P_E^+P_H^- + 2P_E^+P_E^-\}]$$

(Next, we use the fact that $P_H + P_E = 1$, $P_H^+ + P_E^+ = 1$ and $P_H^- + P_E^- = 1$.)

$$= -P_H[\Gamma_{ac} + \Delta\Gamma\{P_H^+(1-P_H^-) + (1-P_H^+)P_H^- + 2(1-P_H^+)(1-P_H^-)\}]$$

$$= -P_H[\Gamma_{ac} + \Delta\Gamma\{P_H^+ - P_H^+P_H^- + P_H^- - P_H^-P_H^+ + 2 - 2P_H^+ - 2P_H^- + 2P_H^+P_H^-\}]$$

$$= -P_H[\Gamma_{ac} + \Delta\Gamma\{2 - P_H^+ - P_H^-\}]$$

$$= -P_H[\Gamma_{ac} + \Delta\Gamma\{P_E^+ + P_E^-\}]$$

Similarly, the second bracket term can be simplified, thereby reducing Eq S16 as,

$$\frac{\partial P_H(x,t)}{\partial t} = -P_H[\Gamma_{ac} + \Delta\Gamma\{P_E^+ + P_E^-\}] + P_E[\Gamma_{me} + \Delta\Gamma\{P_H^+ + P_H^-\}] \quad (S17)$$

Note that if the neighborhood effect is switched off (i.e., $\Delta\Gamma = 0$), Eq S17 reduces to Eq S15 derived above for the case of constant reaction rates. We can simplify Eq S17 further as,

$$\frac{\partial P_H(x,t)}{\partial t} = -\Gamma_{ac}P_H + \Gamma_{me}P_E - \Delta\Gamma[P_H(2 - P_H^+ - P_H^-) - (1-P_H)(P_H^+ + P_H^-)]$$

$$\frac{\partial P_H(x,t)}{\partial t} = \underbrace{[-\Gamma_{ac}P_H + \Gamma_{me}P_E]}_{\text{Reaction-like term}} + \underbrace{\Delta\Gamma[-2P_H + P_H^+ + P_H^-]}_{\text{Diffusion-like term}}$$

To see the diffusion-like similarity of the second, we can expand $P_H^{+/-}$ via Taylor series expansion to obtain,

$$\frac{\partial P_H(x,t)}{\partial t} = \underbrace{[-\Gamma_{ac}P_H + \Gamma_{me}P_E]}_{\text{Reaction-like term}} + \underbrace{\underbrace{(\delta x)^2\Delta\Gamma}_{\substack{\text{Effective}\\ \text{Diffusivity}}}\frac{d^2P_H}{dx^2}}_{\text{Diffusion-like term}} \qquad (S18)$$

Comparing the first term to the Eq S15, we see that it obeys non-conservative reaction-like kinetics, while comparing the second term to Eq S14 we see that it obeys a conservative diffusion-like kinetics. Thus, we immediately see that the evolution decouples naturally into a reaction-like term and a diffusion-like term. This happens purely because the reaction rates are asserted to be neighborhood dependent.

Comparing Eq S18 with the evolution equation used in our model (Eq S10 or Eq 2b) clarifies the mechanistic origin of 'diffusion of epigenetic marks', and proves how epigenetic reaction kinetics influenced by energetics of chromatin-chromatin interactions can give rise to effective diffusion-like kinetics of the epigenetic flavors.

We can use the physical dimensions estimated in Section S9 to get an approximate scale of the energetic parameter ΔE and rate parameter $\Delta\Gamma$. We see later in Section S4, that the diffusion prominently plays a role in the euchromatin region, where in association with the methylation, a reaction-diffusion flux is formed which we predict (see Section S4 for details) will drive the size-scaling of the heterochromatin domains. We estimated the effective diffusivity (see Table S3) to be of the order $\sim 10^{-3}\mu m^2 s^{-1}$. Considering the nucleosome spacing $\Delta x \sim 50$ nm within the euchromatin region, we obtain $\Delta\Gamma \sim 2\ s^{-1}$. Compare this to $\Gamma_{ac} \sim 10^{-2}s^{-1}$, we see that the hetero-to-euchromatin conversion rate increases if the heterochromatin nucleosome is surrounded by one or more euchromatic nucleosomes. In other words, formation of euchromatin would be more stable in the vicinity of other euchromatin. Based on the definition of $\Delta\Gamma$ above, we can approximate $\frac{\Delta\Gamma}{\Gamma_{ac}} \sim e^{(E_{HH}-E_{EH})/k_BT}$. Based on our estimates of $\Delta\Gamma$ and Γ_{ac}, thus we can get $E_{HH} - E_{EH} \sim 5.29k_BT$.

S1.8 Chromatin clustering of STORM images

MATLAB was used for the analysis of STORM images. For chromatin density qualification, Voronoi tessellation-based segmentation was implemented to construct Voronoi polygon of each localization [17]. The corresponding Voronoi polygon of each locus represents a specific region where any points within this region are closer to this locus, such that the size of Voronoi polygon is inversely proportional to the local Voronoi density. Voronoi polygons located at the edge were omitted due to nearly infinite Voronoi area. The Voronoi density map was further constructed through calculating the reciprocal of Voronoi area map. To differentiate between hetero- and eu-chromatic regions, a density-based threshold was applied to filter out low density euchromatic region. The threshold density value was chosen such that 60~100 percentile of the Voronoi density distribution were classified as heterochromatin in the control group. The same density threshold was applied to all other treatments in comparison with the control. The remaining heterochromatin points cloud was then clustered using Density-based spatial clustering of applications with noise (DBSCAN) algorithm [18]. The heterochromatic point clouds were clustered into separated subdomains, such that each subdomain includes all the neighboring and connected Voronoi polygons which are above threshold. The hyperparameters of DBSCAN were set such that the minimal scan points are greater or equal to the dimension of dataset plus 1, namely 3 in our case [18].

S1.9 Quantitative analysis of chromatin distribution in STORM images

Chromatin clusters obtained after the DBSCAN subdomain classification were categorized as LADs and non-LADs depending on proximity to the boundary of the nucleus image. The nucleus shape was detected using boundary detecting algorithm. A characteristic radius of nucleus (R) was then calculated. The minimal distance between each heterochromatin subdomain and nucleus boundary was calculated, such that any subdomain having a distance smaller than $0.15R$ is classified as LADs domain [19].

To quantify the size of non-LAD heterochromatin subdomains, the area of each non-LAD domain was calculated through detecting the boundary of point clouds which gives a polygon enveloping it. An approximating domain radius was calculated by assuming the subdomain to be a circular shape ($r \approx \sqrt{\text{Domain Area}/\pi}$).

The local LADs thickness was measured by sampling the LAD boundary along the nucleus periphery.

S1.10 ChromSTEM sample preparation, imaging, and reconstruction for BJ Fibroblasts.

BJ cell lines (ATCC Manassas, VA) were cultured in Minimum Essential Media (ThermoFisher Scientific, Waltham, MA,#11095080) at physiological conditions (5% CO 2 and 37 °C). Cells were seeded on 35-mm glass-bottom Petri dishes (MatTek Corp.) until approximately 40-50% confluent and were given at least 24 hours to adhere to the dish before fixation. For ChromSTEM sample preparation, the previously published protocol was adapted [20]. Before fixation, cells were thoroughly rinsed three times in Hank's balanced salt solution without calcium and magnesium (EMS). Cells were fixed using 2.5% EM grade glutaraldehyde, 2% paraformaldehyde, 2 mM $CaCl_2$ in 0.1 M sodium cacodylate buffer, pH = 7.4 at room temperature for 5 minutes and then replaced with fresh fixative and fixed on ice for an hour. The cells were then washed with 0.1 M sodium cacodylate buffer 5 times on the ice. The cells were then incubated in a blocking buffer (10 mM glycine, 10 mM potassium cyanide in 0.1 M sodium cacodylate buffer, pH = 7.4) for 15 minutes, followed by staining with 10 µM DRAQ5™ (Thermo Fisher) and 0.1% saponin solution in 0.1 M sodium cacodylate buffer, pH = 7.4 for 10 minutes. After washing with the blocking buffer twice, the cells were incubated in the blocking buffer on ice before photo-bleaching. During photobleaching on a cold stage using continuous epi-fluorescence illumination (150 W Xenon Lamp) with Cy5 red tilter with a 100x objective for 7 minutes, the cells were incubated in 2.5 mM of 3–5′-diaminobenzidine (DAB) solution (Sigma Aldrich) in 0.1 M sodium cacodylate buffer, pH = 7.4. The cells were washed with 0.1 M sodium cacodylate buffer five times and then stained with reduced osmium solution (EMS) containing 2% osmium tetroxide, 1.5% potassium ferrocyanide, 2 mM $CaCl_2$ in 0.15 M sodium cacodylate buffer, pH = 7.4 for 30 minutes on ice. Then the cells were washed with double distilled water five times on ice. Serial ethanol dehydration (30% ethanol, 50%, 70%, 85%, 95%, 100%x3) was followed by Durcupan resin (EMS) infiltration. An infiltration mixture containing equal proportions of 100% ethanol and Durcupan ™ resin mixture 1 (10 mL Durcupan ™ ACM single component A, M, epoxy resin, 10 mL Durcupan ™ ACM single component B, hardener 964, and 0.15 mL Durcupan ™ ACM single component D) was used to infiltrate cells for 30 minutes at room temperature. Next, an infiltration mixture containing 5 mL 100% ethanol and 10 mL Durcupan ™ resin mixture 1 was used to infiltrate the cells for 2 hours at room temperature. Durcupan ™ resin mixture 2 (0.2 mL Durcupan ™ ACM, single component C, accelerator 960 to mixture 1 (10 mL of component A, 10 mL of component B, and 0.15 mL of component D) was used to infiltrate the cells at 50°C in the dry oven for 1 hour. The photobleached

cells were embedded flat with Durcupan™ resin mixture 2 in beem capsules and further cured at 60 °C in the dry oven for 48 hours. An ultramicrotome (UC7, Leica) was used to section 100 nm thick slices that were deposited onto a copper slot grid with carbon/Formvar film. Then, 10 nm colloidal gold fiducial markers were carefully deposited on both sides of the sample. A 200 kV cFEG STEM (HD2300, HITACHI) with HAADF mode was used and while keeping the field of view constant, the sample was tilted from − 60° to 60° with 2° increments on two roughly perpendicular axes. The fiducial markers were used to align the tilt series in IMOD [21] and reconstructed using TomoPy [22] with a penalized maximum likelihood for 40 iterations independently.

S1.11 Domain Center Mapping and Statistical Analysis

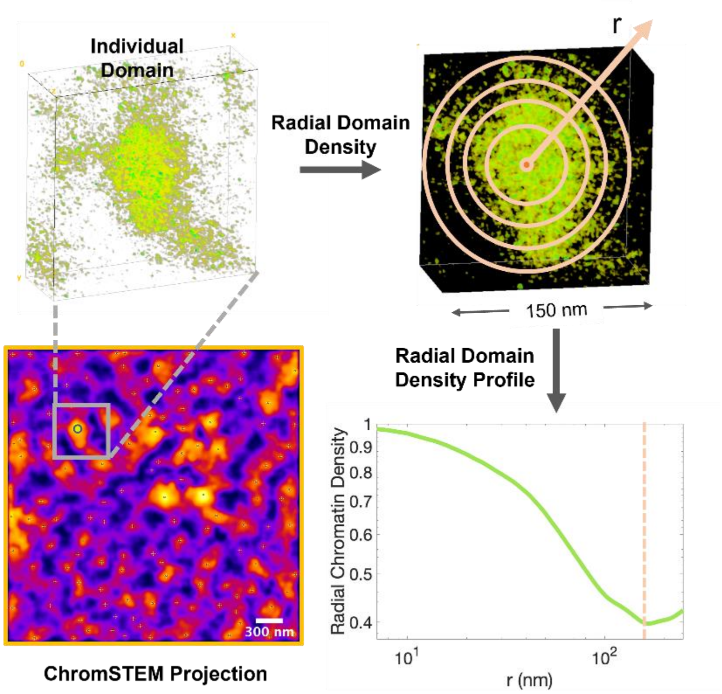

Figure S5: Radial density analysis to establish the radial profile of chromatin packing density estimated as grayscale ChromSTEM intensity within concentric circles with increasing distances from the domain center. n = 1 nuclei with 71 domains.

The centers for individual chromatin domains were estimated from local maxima obtained from ChromSTEM projection with enhanced contrast in FIJI as previously described [23]. Domains occupying less than 50% volume in the z-plane were excluded from the analysis as they could be incomplete parts of other neighboring domains. Mass scaling analysis and radial density analysis were then performed originating from the identified individual domain centers. Average mass scaling originating from the individual domain centers was estimated using the area (mass) weighted by the grayscale ChromSTEM intensity within concentric circles with increasing distances from the domain centers. Similarly, radial chromatin density was estimated as the grayscale ChromSTEM intensity within concentric circles with increasing distances from the domain centers (Figure S5). We have shown that the radial mass density originating from the domain center decreases with increasing distance and approaching the domain boundary and then increases as the boundary of neighboring domains starts interacting. Both the mass scaling behavior and the radial chromatin density profile for each domain were then utilized to obtain the boundary or the approximate radius of the domain. The mass scaling approximately follows power-law scaling up to a given length scale and can be represented by a given slope or scaling exponent, D based on the linear regression of the mass scaling curve in the log-log scale. Beyond the domain regime, the slope gradually increases to reach the supra-domain regime. The radius of chromatin packing domains was estimated as the smallest length scale where the mass scaling curve deviates from the initial power law calculated from small length scales by 5% and the radial chromatin density starts to increase after a gradual decrease. The distributions for packing domain radius and density are shown as mean ± S.D. using violin super plots [24].

S1.12 ActD Treatment for PWS and ChromSTEM imaging

Prior to imaging, cells were cultured in 35-mm glass-bottom Petri dishes until approximately 70% confluent. All cells were given at least 24 hours to re-adhere before treatment (for treated cells) and imaging. HCT116, A549, HeLa, and U2OS cells were treated with Actinomycin D (Gibco, Cat: 11805017) for 1 hour at a final concentration of 5 µg/mL.

S1.13 PWS image acquisition and approximation of domain size scales

For live-cell measurements, cells were imaged and maintained under physiological conditions (5% CO2 and 37°C) using a stage-top incubator (In Vivo Scientific, Salem, SC; Stage Top Systems).

The PWS optical instrument was built on a commercial inverted microscope (Leica, Buffalo Grove, IL, DMIRB) supplemented with a Hamamatsu Image-EM CCD camera C9100-13. This camera was coupled to an LCTF (CRi Woburn, MA) for hyperspectral imaging. Spectrally resolved images of live cells were collected between 500 and 700 nm with a 2-nm step size. Broadband illumination was provided by an Xcite-120 light-emitting diode lamp (Excelitas, Waltham, MA). PWS is a high-throughput, label-free approach that measures the spectral standard deviation (Σ) of internal optical scattering originating from nuclear chromatin. The variations in the refractive index distribution Σ, are characterized by a mass density autocorrelation function (ACF) to calculate chromatin packing, scaling *D*.

Based on ChromSTEM [23], we have previously reported that chromatin packs into domains, wherein each domain exhibits a polymeric fractal-like behavior and can be described by an average packing scaling exponent. This implies that within the fractal regime, the genomic size of chromatin scales with its physical size following a power law relationship. Therefore, we estimated the upper bound of the power law regime as a measure of domain size.

Thus, a power-law ACF which incorporates a lower and upper length scale limit of the power law regime was utilized for the subsequent approximations,

$$B_\rho(r, D_B, l_{min}, l_{max}) = \sigma_\rho^2 \frac{D_B - 3}{l_{max}^{D_B-3} - l_{min}^{D_B-3}} r^{D_B-3} \left[\Gamma\left(\frac{r}{l_{max}}, D_B - 3\right) - \Gamma\left(\frac{r}{l_{min}}, D_B - 3\right)\right]$$

where $\Gamma(x,a)$ is the upper incomplete gamma function, and $l_{\min}$ and $l_{\max}$ characterize fractality's lower and upper length scales, respectively; $B_\rho(r=0)$ is $\sigma\rho^2$, the variance of chromatin mass density; D_B describes the shape of $B\rho$ and is related to D, and *r* is the spatial separation Utilizing this previously described methodology [25], we evaluated $l_{\max}$, the upper length scale of chromatin mass density scaling to estimate the relative size of domains upon ActD treatment.

S2 Heterochromatin domain morphology dependence on Epigenetic Rates

Our model predicts that the heterochromatin domains obtained at the steady state display a characteristic size. The length scale of the stable domains is regulated in tandem by the epigenetic reactions – acetylation as well as methylation – and the transcriptionally active extrusion of chromatin loops. As the levels of histone acetylation are increased (or conversely methylation is decreased), as shown in Figure S6a, we see that the sizes of the heterochromatin domains

decrease. This trend is displayed not just by the interior heterochromatin domains, but also by the LADs near the nuclear periphery.

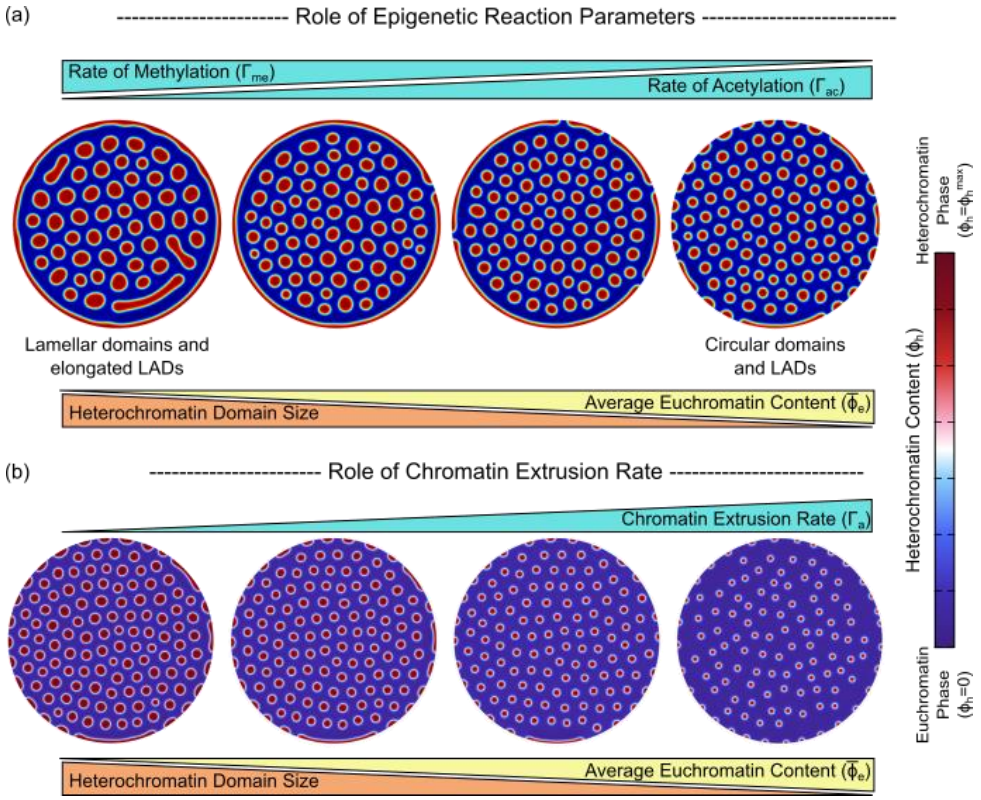

Figure S6: A study of the effect of reaction parameters (a) for epigenetic regulation and (b) rate of chromatin extrusion governed by transcription.

Also note that the morphology of the heterochromatin domains is regulated by the balance of acetylation and methylation reaction kinetics. Under the conditions where acetylation predominates over methylation, the domains formed are more circular. On the other hand, when methylation is increased while acetylation is reduced, the domains become larger and predominantly lamellar.

In addition to methylation and acetylation, transcriptional activity also regulates the sizes of the heterochromatin domains (Figure S6b). As the rate of supercoiling-driven chromatin extrusion reduces, the heterochromatin domains become larger in size. This is discussed expansively in Sections 3.2 and 3.3 of the main manuscript (also see Figure 2d-g).

S3 Theoretical analysis of average chromatin phase contents determined by reactions

Here we show that the total amount of chromatin which falls into the individual phases, i.e., either euchromatin or heterochromatin, is determined solely by the epigenetic and chromatin extrusion reaction kinetics. The spatiotemporal evolution of order parameter ϕ_d due to the presence of diffusion of epigenetic marks and reaction driven interconversion of eu- and heterochromatin phases, as described by Eq 2b (or equivalently, Eq S10) as,

$$\frac{\partial \phi_d}{\partial t} = M_d \nabla^2 \mu_d + 2(\Gamma_{me}\phi_e - \Gamma_{ac}\phi_h) - 2\Gamma_a(\boldsymbol{x})\phi_h \qquad (S19)$$

Once steady state is reached, the evolution halts and $\frac{\partial \phi_d}{\partial t} = 0$. At this stage, the relation between the average euchromatin content $\bar{\phi}_e$ and the average heterochromatin $\bar{\phi}_h$ content in the nucleus can be obtained by averaging Eq S19 (i.e., integrating over the entire nucleus or region of interest and divide by the area of the nucleus) as,

$$-M_d \frac{\int_\Omega \nabla^2 \mu_d \, dV}{\int_\Omega dV} = 2\Gamma_{me}\bar{\phi}_e - 2\Gamma_{ac}\bar{\phi}_h - \frac{2}{\int_\Omega dV}\int_\Omega \Gamma_a(\boldsymbol{x})\phi_h(\boldsymbol{x})dV$$

Note that the term $\nabla^2 \mu_d$ on the left-hand side is non-zero near the domain boundaries and after averaging over the entire volume of the nucleus can be approximated to zero. Thus,

$$0 = 2\Gamma_{me}\bar{\phi}_e - 2\Gamma_{ac}\bar{\phi}_h - \frac{2}{\int_\Omega dV}\int_\Omega \Gamma_a(\boldsymbol{x})\phi_h(\boldsymbol{x})dV$$

The last term on the right-hand side can be resolved via integration by parts as,

$$\frac{\int_\Omega \Gamma_a e^{-\left(\frac{\phi_h - \frac{\phi_h^{max}}{2}}{2\,\Delta\phi}\right)^2} \phi_h(\boldsymbol{x})dV}{\int_\Omega dV}$$

$$= \Gamma_a e^{-\left(\frac{\phi_h - \frac{\phi_h^{max}}{2}}{2\,\Delta\phi}\right)^2} \frac{\int_\Omega \phi_h(\boldsymbol{x})dV}{\int_\Omega dV} - \frac{\Gamma_a}{\int_\Omega dV}\int_\Omega \phi_h(\boldsymbol{x})\left(-\frac{\phi_h - \frac{\phi_h^{max}}{2}}{\Delta\phi}\right) e^{-\left(\frac{\phi_h - \frac{\phi_h^{max}}{2}}{2\,\Delta\phi}\right)^2} dV$$

The last term (in blue) when integrated over the domain measures the length of the interfaces between the heterochromatin and euchromatin domains. The term in red is non-zero only along the interface. For a narrow interface width $\Delta\phi \to 0$, this can be approximated as multiplying the integrand by a factor depending on the length of the interface giving a parameter ℓ_{int}. Thus,

$$\frac{\int_\Omega \Gamma_a e^{-\left(\frac{\phi_h - \frac{\phi_h^{max}}{2}}{2\,\Delta\phi}\right)^2} \phi_h(\boldsymbol{x})dV}{\int_\Omega dV} \approx \Gamma_a e^{-\left(\frac{\phi_h - \frac{\phi_h^{max}}{2}}{2\,\Delta\phi}\right)^2} \bar{\phi}_h(\ell_{int}) = \Gamma_a \bar{\phi}_h \kappa$$

Thus, the equation can be written as,

$$0 = 2\Gamma_{me}\bar{\phi}_e - 2\Gamma_{ac}\bar{\phi}_h - 2\Gamma_a\bar{\phi}_h\kappa$$

κ being a non-trivial function dependent on ϕ_h^{max}, volume fraction change across the interface $\Delta\phi$, and the length of the interface between the two chromatin phases. By definition of the volume fractions, $\phi_e = 1 - \phi_n - \phi_h$. Thus,

$$(\Gamma_{me} + \Gamma_{ac} + \kappa\Gamma_a)\bar{\phi}_h = \Gamma_{me}(1 - \bar{\phi}_n)$$

Or,

$$\bar{\phi}_h \approx \frac{\Gamma_{me}\,(1 - \bar{\phi}_n)}{\Gamma_{me} + \Gamma_{ac} + \kappa\Gamma_a}, \qquad \bar{\phi}_e \approx \frac{(\Gamma_{ac} + \kappa\Gamma_a)\,(1 - \bar{\phi}_n)}{\Gamma_{me} + \Gamma_{ac} + \kappa\Gamma_a} \qquad (S20)$$

In the absence of transcription ($\Gamma_a = 0$), we can instead write the average heterochromatin and euchromatin contents as,

$$\bar{\phi}_h \approx \frac{\Gamma_{me}\,(1-\bar{\phi}_n)}{\Gamma_{me}+\Gamma_{ac}}, \qquad \bar{\phi}_e \approx \frac{\Gamma_{ac}\,(1-\bar{\phi}_n)}{\Gamma_{me}+\Gamma_{ac}} \tag{S21}$$

The average eu- and heterochromatin contents obtained via Eq S20 and S21 can be rescaled to obtain:

$$\bar{\phi}_h \approx \frac{\tilde{\Gamma}_{me}\,(1-\bar{\phi}_n)}{\tilde{\Gamma}_{me}+1+\kappa\tilde{\Gamma}_a}, \qquad \bar{\phi}_e \approx \frac{(1+\kappa\tilde{\Gamma}_a)\,(1-\bar{\phi}_n)}{\tilde{\Gamma}_{me}+1+\kappa\tilde{\Gamma}_a} \tag{S22}$$

$$\bar{\phi}_h \approx \frac{\tilde{\Gamma}_{me}\,(1-\bar{\phi}_n)}{\tilde{\Gamma}_{me}+1}, \qquad \bar{\phi}_e \approx \frac{(1-\bar{\phi}_n)}{\tilde{\Gamma}_{me}+1} \tag{S23}$$

S4 Domain size determination in presence of transcription – theoretical analysis

The steps involved in theoretical derivation of heterochromatin domain size have been enumerated in the main text (Section 3.2). Here we show the complete derivation of domain size determination in the interior of the nucleus, away from the periphery. To analyze the steady state size of the heterochromatin domain, we first examine the volume fraction fields within and around the droplet. As discussed in Section S3, the acetylation, methylation and chromatin extrusion together determine the mean heterochromatin (and euchromatin) volume fraction in the nucleus, given by Eq S20. In absence of any energetic considerations, this would give rise to a homogeneous mean chromatin composition ($\bar{\phi}_h, \bar{\phi}_e$). However, this composition (Figure 2b; light blue circle) lies in neither of the energy wells and is thus energetically unfavorable. As the system marches towards a steady state, its free energy must reduce, requiring the phase separation to initiate via nucleation of heterochromatin droplets (Figure S7).

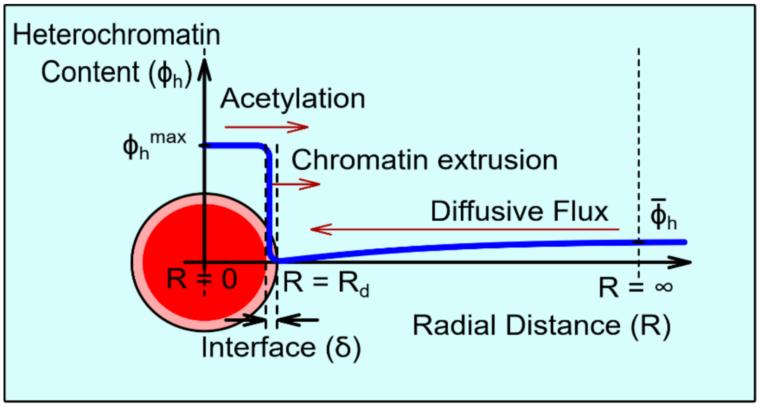

Figure S7: The competition of diffusion driven influx of heterochromatin with the epigenetic reaction and transcription mediated extrusion driven outflux of heterochromatin from the heterochromatin domain determines its steady state size. The figure also shows the radial distribution of heterochromatin volume fraction ϕ_h in and around the domain.

Under a dilute limit, i.e., when there is a lot more euchromatin than heterochromatin, we can assume that the droplet size is much smaller than the length scale of the interdomain spacing such that neighboring droplets are far enough to not interact with each other. Under such assumption the heterochromatin distribution would be spherically symmetric. Using a polar coordinate system with origin at the droplet center, we can determine radial distribution of the heterochromatin volume fraction $\phi_h(R)$ as,

$$\phi_h(R) = \begin{cases} \phi_h^{max} & R < R_d & \text{(driven by phase-separation)} \\ \phi_h^+ = \dfrac{\eta}{R} & R = R_d^+ & \text{(driven by interfacial surface tension)} \\ \bar{\phi}_h & R = \infty & \text{(region far from the droplet stays undisturbed)} \end{cases}$$

where R_d is the radius of the droplet at the current instance, while η measures the interfacial energy. Figure S7 shows the distribution of heterochromatin volume fraction $\phi_h(R)$ around a single spherical domain of heterochromatin (red) of radius R_d as it grows surrounded by euchromatin phase (in blue). The slope of the heterochromatin profile outside the droplet will drive an inward flux of heterochromatin into the droplet. The volume fraction field outside the droplet at steady state must follow the equation,

$$0 = D_h \nabla^2 \phi_h - (\Gamma_{ac} + \Gamma_a)\phi_h + \Gamma_{me}\phi_e$$

with boundary conditions $\phi_h|_{R_d^+} = \eta/R$ and $\phi_h|_\infty = \bar{\phi}_h$, and thus must have the form,

$$\phi_h^{out}(R) = \bar{\phi}_h + (\phi_h^+ - \bar{\phi}_h)\frac{R_d}{R} e^{\frac{R_d - R}{\ell_{RD}}}$$

where ℓ_{RD} is the characteristic reaction-diffusion length scale given under a dilute limit as $\ell_{RD} = \sqrt{\frac{D_h}{\Gamma_{ac}}}$. Note that the transcription driven active extrusion occurs only at the periphery and thus does not play a role in the reaction-diffusion length scale. Thus,

$$\frac{\partial \phi_h^{out}}{\partial R} = (\bar{\phi}_h - \phi_h^+)\frac{R_d(\ell_{RD} + R)}{\ell_{RD} R^2} e^{\frac{R_d - R}{\ell_{RD}}}$$

Thus,

$$\left.\frac{\partial \phi_h^{out}}{\partial R}\right|_{R \to R_d} = \frac{\bar{\phi}_h}{R_d} - \frac{\eta}{R_d^2} \qquad \left(when \frac{R_d}{\ell_{RD}} \ll 1\right) \qquad (S24)$$

At the periphery of the droplet, the diffusive influx of heterochromatin into the droplet due to the reaction-diffusion phenomena outside is,

$$J^{in} = 4\pi R_d^2 D_h \left.\frac{\partial \phi_h^{out}}{\partial R}\right|_{R \to R_d}$$

The inward diffusion is opposed by the outward flux of heterochromatin into euchromatin phase which occurs due to both acetylation of histones inside as well as supercoiling-driven chromatin extrusion along the domain boundary. Thus, the rate of change of the volume of the droplet V_d can be written as,

$$\frac{dV_d}{dt} = \underbrace{J^{in}}_{\text{inwards diffusion}} - \underbrace{\Gamma_{ac} \times \frac{4}{3}\pi R_d^3 \phi_h^{max}}_{\text{Acetylation}} - \underbrace{4\pi R_d^2 \ell_{int} \Gamma_a \times \frac{\phi_h^{max}}{2}}_{\text{Chromatin extrusion}}$$

where ℓ_{int} is the width of the interface between the chromatin phases. Simplifying this we obtain,

$$4\pi R_d^2 \frac{dR_d}{dt} = 4\pi D_h(\bar{\phi}_h R_d - \eta) - \Gamma_{ac} \times \frac{4}{3}\pi R_d^3 \phi_h^{max} - 4\pi R_d^2 \frac{\ell_{int}}{2} \Gamma_a \phi_h^{max}$$

$$\frac{dR_d}{dt} = D_h\left(\frac{\bar{\phi}_h}{R_d} - \frac{\eta}{R_d^2}\right) - \frac{\Gamma_{ac}R_d}{3}\phi_h^{max} - \frac{\ell_{int}}{2}\Gamma_a\phi_h^{max} \quad (S25)$$

We reproduce Eq S25 in the main text as Eq 4 for a small value of interfacial energy ($\eta \to 0$). Using Eq S25 to plot the rate of change of heterochromatin domain size with respect to the instantaneous domain radius we obtain the plot shown in Figure S8.

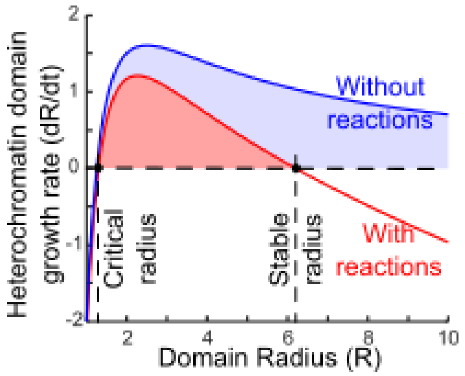

Figure S8: The growth rate of the heterochromatin domain varying with its instantaneous radius.

Above a critical radius, all heterochromatin domains growth in size ($dR_d/dt > 0$). In the absence of reactions ($\Gamma_{ac} = \Gamma_a = 0$, blue curve), we note that the rate of increase in the heterochromatin domain radius is always positive indicating that the domain will keep growing as long as its radius is larger than the critical radius (See section S5 for further discussion). However, in the presence of the reactions, we note that the domains will grow until their growth rate reaches a zero value. This gives the stable size of heterochromatin domains i.e., domains which neither grow nor shrink. The domains larger than the stable radius will shrink back to the stable radius.

The stable radius (R_d^{ss}) can be obtained by setting $dR_d/dt = 0$ in Eq S25 such that,

$$\frac{\Gamma_{ac}}{3}R_d^{ss\,2} + \frac{\Gamma_a\ell_{int}}{2}R_d^{ss} - \frac{D_h\bar{\phi}_h}{\phi_h^{max}} = 0 \quad (S26)$$

Thus,

$$R_d^{ss} = \frac{-\frac{\Gamma_a\ell_{int}}{2} + \sqrt{\left(\frac{\Gamma_a\ell_{int}}{2}\right)^2 + \frac{4}{3}\frac{D_h}{\phi_h^{max}}\frac{\Gamma_{ac}\Gamma_{me}(1-\bar{\phi}_n)}{\Gamma_{me}+\Gamma_{ac}+\kappa\Gamma_a}}}{2\frac{\Gamma_{ac}}{3}}$$

$$R_d^{ss} = -\frac{3\Gamma_a\ell_{int}}{4\Gamma_{ac}} + \sqrt{\left(\frac{3\Gamma_a\ell_{int}}{4\Gamma_{ac}}\right)^2 + \frac{3D_h}{\Gamma_{ac}\phi_h^{max}}\frac{\frac{\Gamma_{me}}{\Gamma_{ac}}(1-\bar{\phi}_n)}{1+\frac{\Gamma_{me}}{\Gamma_{ac}}+\kappa\frac{\Gamma_a}{\Gamma_{ac}}}} \quad (S27)$$

Note that in the absence of transcription, the steady state domain size can be obtained by substituting $\Gamma_a = 0$ as,

$$R_d^{ss}|_{\Gamma_a=0} = \sqrt{\frac{3D_h}{\Gamma_{ac}\phi_h^{max}}\frac{\frac{\Gamma_{me}}{\Gamma_{ac}}(1-\bar{\phi}_n)}{1+\frac{\Gamma_{me}}{\Gamma_{ac}}}} \quad (S28)$$

The interior heterochromatin radii in the nuclei treated with ActD must approximately follow Eq S28. Further, we have assumed a dilute limit for the analytical derivations, which implies that the

neighboring heterochromatin domains do not interact with each other. Such assumption therefore requires that the domains be separated by a distance which scales with the reaction-diffusion length scale such that the spacing between the domains can be written as,

$$S_d^{ss}|_{\Gamma_a=0} = \sqrt{\frac{3D_h}{\Gamma_{ac}}} \qquad (S29)$$

Thus, a quantitative image analysis of super-resolution images of heterochromatin foci in in-vitro nucleus can be used to quantitatively estimate the parameters Γ_{ac} and Γ_{me} using Eq S28. As discussed in Section S6, we will use these relations to motivate the parameter choice for our numerical simulations.

For consistency with our non-dimensional model, we can rescale Eq S27-29 to obtain a non-dimensional dependence of heterochromatin domain size on the epigenetic and transcriptional kinetics. All lengths are rescaled with respect to ℓ_{RD}, while all times with respect to $1/\Gamma_{ac}$. Thus, Eq S27 becomes,

$$\tilde{R}_d^{ss} = -\frac{3\tilde{\Gamma}_a\delta}{4} + \sqrt{\left(\frac{3\tilde{\Gamma}_a\delta}{4}\right)^2 + \frac{3}{\phi_h^{max}}\frac{\tilde{\Gamma}_{me}(1-\bar{\phi}_n)}{1+\tilde{\Gamma}_{me}+\kappa\tilde{\Gamma}_a}} \qquad (S30)$$

While Eq S28 and S29 become,

$$\tilde{R}_d^{ss}|_{\tilde{\Gamma}_a=0} = \sqrt{\frac{3\tilde{\Gamma}_{me}}{\phi_h^{max}}\frac{(1-\bar{\phi}_n)}{1+\tilde{\Gamma}_{me}}} \qquad (S31)$$

$$\tilde{S}_d^{ss}|_{\tilde{\Gamma}_a=0} = \sqrt{3} \qquad (S32)$$

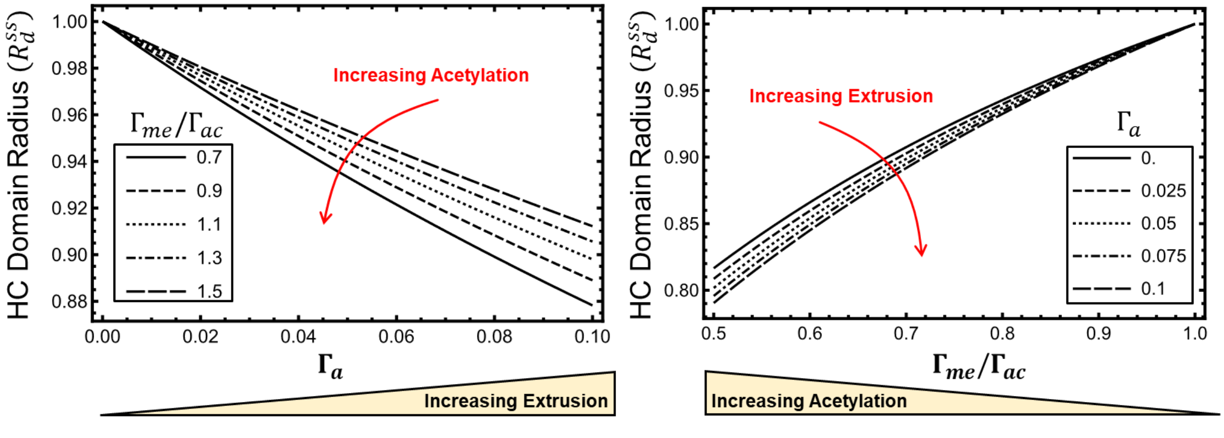

Figure S9: Change in domain radius $\tilde{R}_d^{ss}$ as rate of acetylation Γ_{ac} or rate of chromatin extrusion $\tilde{\Gamma}_a$ increase.

Note that although the qualitative effect of increasing acetylation (or decreasing methylation) is the same as increasing rate of supercoiling-drive chromatin extrusion, the quantitative ways in which their effects are felt are different (different scales in Eq S25, S26). This is immediately visible from Eq S30 and S31. With $\tilde{\Gamma}_{me} = \Gamma_{me}/\Gamma_{ac}$, the domain radius scales as, $\tilde{R}_d^{ss} \sim \sqrt{\frac{\tilde{\Gamma}_{me}}{1+\tilde{\Gamma}_{me}}}$. On the other hand, the scaling of domain radius with $\tilde{\Gamma}_a$ is more complex, but if the effect of acetylation

is ignored, from Eq S26, we see that $\tilde{R}_d^{ss} \sim \frac{1}{\tilde{\Gamma}_a}$. This difference in scales can be exemplified by graphically seeing the change in $\tilde{R}_d^{ss}$ as these rates change, as shown in Figure S9. We see that the effect of acetylation is more pronounced than the effect of chromatin extrusion. However, this result is derived theoretically for a single domain. Since a single domain has only one continuous boundary where transcription occurs, this scaling holds. In a numerical simulation, there are many boundaries where transcription will drive chromatin extrusion, and thus change the scaling derived theoretically. The numerical effect of changing epigenetic reaction rate, and extrusion rate was discussed in Section S2, Figure S5. Theoretically this can be captured by increasing the value of κ in Eq S30. The parameter κ, as discussed in Section S3, includes, amongst other effects, the role of multiple domain boundaries.

S5 A characteristic size of heterochromatin domains is not obtained without reactions

As discussed in Section 3.1, the steady state organization of chromatin the nucleus (Figure 2) comprises of many disconnected domains of heterochromatin phase ($\phi_h = \phi_h^{max}$) with a characteristic size. We also determined that the size of these domains is determined by the reaction kinetics – the rates of acetylation Γ_{ac}, methylation Γ_{me} and active chromatin extrusion Γ_a (Eq 5).

To investigate the role of reactions numerically, we allow the phase-separation to occur from the same initial state as in Figure 2a, but without any reaction kinetics. The initial, intermediate and steady state chromatin organization thus obtained is shown in Figure S10a. While intermediate steps show the nucleation of multiple domains, much like in the presence of reactions (Figure 2a, center panel). However, as the organization evolves these domains merge. At the steady state, a single domain of heterochromatin remains. The growth rate of the domains given by Eq 4 is plotted in Figure S10b. In the absence of reactions, dR_d/dt never goes to zero, except at the critical radius. The critical radius only ensures that the domains above this size grow, while the rest shrink. Since no stable radius is predicted, the growing domains continue to grow $\left(\frac{dR_d}{dt} > 0\right)$, until all heterochromatin merge into a single domain. This leads to full Ostwald ripening.

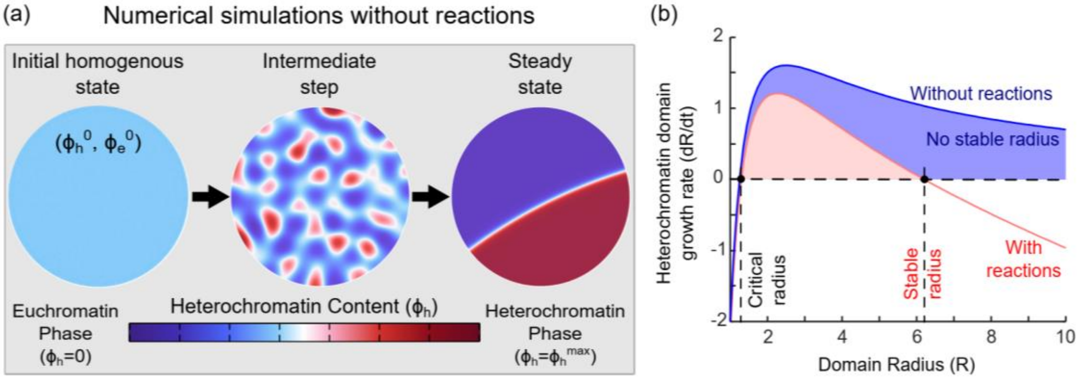

Figure S10: Steps in the numerical simulation show the evolution of chromatin organization in absence of the epigenetic and transcriptionally mediated reaction kinetics. While nucleation of multiple domains does occur, as they evolve all the nucleated domains merge into a single heterochromatin domain. (b) Plot of theoretically evaluated growth rate of heterochromatin domains with (red) and without (blue) reactions. Reactions give rise to a stable domain radius. In the absence of reactions, there is no stable domain radius.

S6 Stable domain radius is not significantly regulated by interfacial effects

We have seen via the derivation in Section S4 and Eq S25 that a steady state heterochromatin domain (obtained by setting $dR/dt = 0$) is regulated via reaction kinetics, and apparently by the interfacial energy penalty η. Here we show that the contribution of η in determining R_d^{ss} is very small.

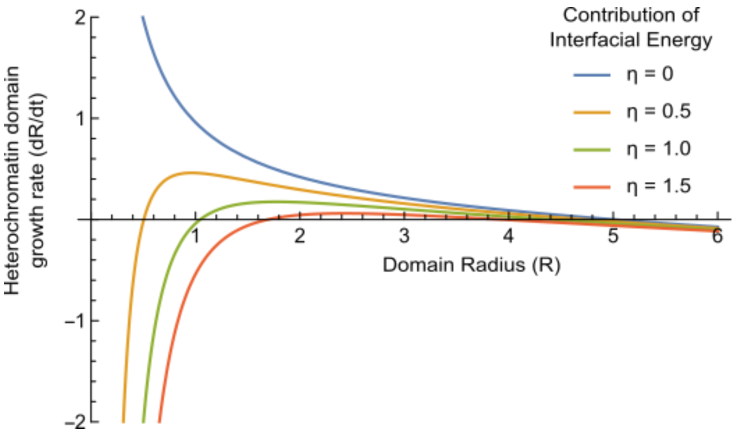

Figure S11: Effect of interfacial energy penalty η on the stable radius of heterochromatin domains.

As in the previous section, we plot the growth rate of the heterochromatin droplets with respect to their current radius, as obtained from Eq S25 (Figure S11). The stable radius of the domains R_d^{ss} is the point where the curve intersects with the x-axis such that the growth rate of the domains becomes zero, i.e. $dR_d/dt = 0$. As the contribution of the interfacial energy is changed by changing the energetic penalty η on the formation of interfaces, we note that the change in the domain sizes is very small. Even when $\eta = 0$, we see that the domain size does not change appreciably.

These results highlight a key role played by the epigenetic reactions as well as transcriptional regulation of chromatin extrusion. Interestingly, these results also highlight the key difference between our 'non-equilibrium' thermodynamic phase-separation model for chromatin organization as opposed to a more traditional energy-minimizing phase-separation. The traditional phase-field models result in domain formation by a competition between the energy reducing phase separation and energy increasing interface formation. However, in our model, the role of the interface formation is overshadowed by the role of reactions in a kinetics rather than energetics driven formation and maintenance of heterochromatin domains. This 'competition' between the energetic phase-separation and kinetics of interconverting reactions results in formation of heterochromatin domains of characteristic sizes.

S7 LAD thickness determination in presence of transcription – theoretical analysis

Like the determination of the radius of the heterochromatin droplets in the interior of the nucleus, at the nuclear periphery, the epigenetic, transcriptional and diffusion kinetics balance regulates the thickness of the LADs.

We begin by examining the volume fraction fields within and around the LAD. As discussed in Section S4, the acetylation, methylation and chromatin extrusion together determine the mean

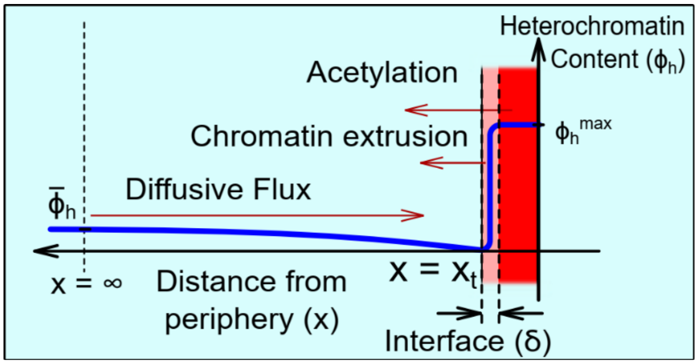

Figure S12: The competition of diffusion driven influx of heterochromatin with the epigenetic reaction and transcription mediated extrusion driven outflux of heterochromatin from the heterochromatin domain determines its steady state size. The figure also shows the radial distribution of heterochromatin volume fraction ϕ_h in and around the domain.

heterochromatin (and euchromatin) volume fraction in the nucleus, given by Eq S20. However, a homogenous mean chromatin composition $(\bar{\phi}_h, \bar{\phi}_e)$ lies in neither of the energy wells as shown in Figure 2b (light blue circle) and is thus energetically unfavorable. Nucleation of heterochromatin domains occurs due to the reduction of free energy as the system evolves. The interaction of heterochromatin with the nuclear lamina results in a preferential nucleation of heterochromatin domains along the lamina i.e., lamina associated domains, LADs. We assume that the LADs are formed uniformly along the lamina, and can grow normal to the lamina i.e., increase in thickness, as shown in Figure S12. We also assume that the LADs are far away from the neighboring interior heterochromatin domains, and do not interact with them.

Using a cartesian coordinate system with origin at the nuclear lamina directed normal to it, we determine spatial distribution of the heterochromatin volume fraction $\phi_h(x)$. Note that this can be easily done by extending the derivations for the interior heterochromatin domains by setting $R \to \infty$, to obtain a linear continuous LAD. Thus,

$$\phi_h(x) = \begin{cases} \phi_h^{max} & x < x_t & \text{(driven by phase-separation)} \\ \phi_h^{+} \to 0 & x = x_t^{+} & \text{(set R}\to\infty\text{, interfacial contribution)} \\ \bar{\phi}_h & x = \infty & \text{(region far from the LAD stays undisturbed)} \end{cases}$$

where $x_t(t)$ is the thickness of the LAD at any time-step. Figure S12 shows the distribution of heterochromatin volume fraction $\phi_h(x)$ in the vicinity of a LAD (in red) of thickness x_t as it grows surrounded by euchromatin phase (in blue). The volume fraction field outside the droplet at steady state must follow the evolution equation,

$$0 = D_h \nabla^2 \phi_h - (\Gamma_{ac} + \Gamma_a)\phi_h + \Gamma_{me}\phi_e$$

with boundary conditions $\phi_h|_{x_t^+} = 0$ and $\phi_h|_{x=\infty} = \bar{\phi}_h$, and thus must have the form,

$$\phi_h^{out}(x) = \bar{\phi}_h\left(1 - e^{-(x-x_t)/\ell_{RD}}\right)$$

where ℓ_{RD} is the characteristic reaction-diffusion length scale given under a dilute limit as $\ell_{RD} = \sqrt{\frac{D_h}{\Gamma_{ac}}}$. The slope of the heterochromatin profile outside the droplet will drive an inward flux of heterochromatin into the droplet. As for the interior heterochromatin domains, the inward diffusion is opposed by the outward flux of heterochromatin into euchromatin phase which occurs due to both acetylation of histones inside as well as DNA loop extrusion along the domain periphery. Thus, the rate of change of the volume of the droplet V_d can be written as,

$$\frac{dV_{LAD}}{dt} = S\frac{dx_t}{dt} = J^{in} - \Gamma_{ac} S x_t \phi_h^{max} - S\ell_{int}\Gamma_a \frac{\phi_h^{max}}{2} \qquad (S33)$$

where $J_{in} = SD_h \frac{\partial \phi_h^{out}}{\partial x}\Big|_{x \to x_t} \sim \frac{SD_h}{l} \bar{\phi}_h$ is the diffusive influx. At steady state, by setting $dx_t/dt = 0$, we obtain,

$$x_t^{ss} = \frac{D_h}{\Gamma_{ac} \ell_{RD} \phi_h^{max}} \frac{\Gamma_{me}\,(1 - \bar{\phi}_n)}{\Gamma_{me} + \Gamma_{ac} + \kappa \Gamma_a} - \frac{\Gamma_a}{2\Gamma_{ac}} \ell_{int}$$

$$x_t^{ss} = \frac{\ell_{RD} \Gamma_{me} (1 - \bar{\phi}_n)}{\phi_h^{max} (\Gamma_{me} + \Gamma_{ac} + \kappa \Gamma_a)} - \frac{\Gamma_a}{2\Gamma_{ac}} \ell_{int}$$

Rescaling,

$$\tilde{x}_t^{ss} = \frac{\tilde{\Gamma}_{me} (1 - \bar{\phi}_n)}{\phi_h^{max} \left(1 + \tilde{\Gamma}_{me} + \kappa \tilde{\Gamma}_a\right)} - \frac{\delta \tilde{\Gamma}_a}{2} \qquad (S34)$$

S8 Model calibration and validation

Having developed the model to capture the spatio-temporal organization of chromatin in the nucleus, we numerically solve Eq S12 and S13. The choice of parameters used in our simulations are motivated by the discussions in this section. The parameters can be broadly classified into four types.

Kinetic parameters: In the non-dimensional model (Eq S12 and S13), all times are rescaled with respect to the timescale of acetylation reaction rate Γ_{ac} (as discussed in the extended methods, Section S1), and hence the only parameters which can be altered are the non-dimensional rates of methylation $\tilde{\Gamma}_{me}$ and active chromatin extrusion $\tilde{\Gamma}_a$. As discussed in section S4, the rates of methylation and acetylation govern the size of the heterochromatin domain and only the acetylation rate determines the intra-domain spacing.

In the rescaled model, the distribution of the heterochromatin domains viz. their sizes is regulated only by $\tilde{\Gamma}_{me} = \Gamma_{me}/\Gamma_{ac}$. The spacing between the domains is regulated by the size of the domain chosen to model the nucleus. We choose the diameter of nucleus much greater than the reaction-diffusion length-scale ($L_{nucleus} \gg \ell_{RD}$). Specifically, we chose the nuclear diameter $\sim 20\ell_{RD}$, such that the simulations give the heterochromatin domain spacing qualitatively similar to the inter-domain spacing observed in-vitro via STORM imaging of ActD-treated nuclei. ActD-treated nuclei are specifically chosen for parameter estimation so as to eliminate the effects of transcription mediated chromatin extrusion rate $\tilde{\Gamma}_a$ in determining the chromatin organization. Next, the size of the heterochromatin domains is controlled by $\tilde{\Gamma}_{me}$. This parameter is obtained quantitatively from the analysis of the images of ActD-treated nuclei so as to get a similar size distribution in the simulation. Also note that the choice of $\tilde{\Gamma}_{me}$ will control the morphology of the heterochromatin domains (discussed in Section S2, Figure S4). We choose $\tilde{\Gamma}_{me}$ so as to obtain predominantly nearly circular domains so as to facilitate a more straight-forward calculation of heterochromatin domain length-scales. The calibrated values for $L_{nucleus}$ and $\tilde{\Gamma}_{me}$ are listed in Table S2. A qualitatively comparable prediction of chromatin organization upon transcription inhibition as well as under control conditions validates our parameter choice (Section 3.3). Note that all the simulations reported in the main manuscript as well as the SI use the same values for $L_{nucleus}$ and $\tilde{\Gamma}_{me}$. Chromatin extrusion rate Γ_a is calibrated and validated based on in-vitro nuclear imaging as discussed in Section 3.3 of the main manuscript and algorithmically depicted in Figure S14.

Energetic parameters: The non-dimensional energy density (Eq 1 or Eq S11) involves a single parameter – the chromatin-lamina interaction strength $\tilde{V}_L$. We have previously [26] shown that, $\tilde{V}_L$ plays a role in deciding the LAD thickness and morphology, i.e. whether the LAD would be more droplet shaped or lamellar. The LAD morphology observed in in-vitro nuclei with transcriptional abrogation is used to estimate the value of $\tilde{V}_L$. For the previously estimated values of epigenetic reaction rates, we parametrically vary $\tilde{V}_L$ (Figure S13) to obtain a close match with the LAD distribution in ActD treated nucleus (Figure 3d, right panel) as reported in Table S2. All the simulations reported here use the same value for $\tilde{V}_L$, hence validating its choice with the cases where transcription is active.

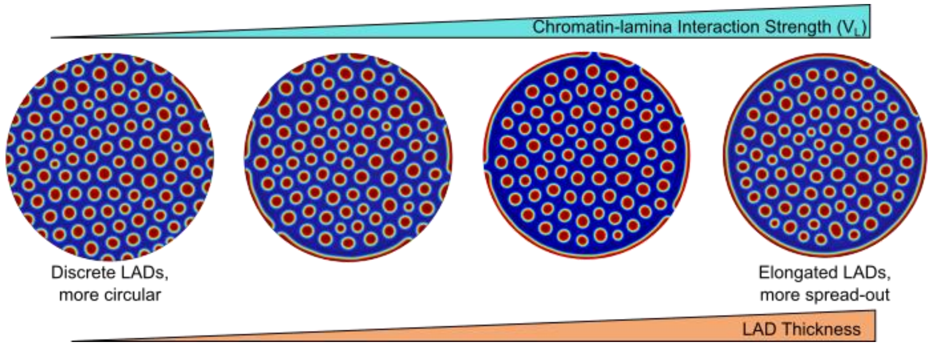

Figure S13: Results of a parametric study on the variation of LAD thickness as the chromatin-lamina interaction strength $\tilde{V}_L$ is increased. As $\tilde{V}_L$ increases, the LADs become more spread out over the entire nuclear periphery. A comparison with distribution of LAD observed in in-vitro nuclei allows the evaluation of the parameter $\tilde{V}_L$. Further note that change in $\tilde{V}_L$ has no effect on the sizes of the heterochromatin domains in the interior of the nucleus.

Initial/boundary conditions: We consider an initial spatially homogenous distribution of chromatin and nucleoplasm in the nucleus. The nucleoplasm content of the nucleus is estimated based on experimental images as $\phi_n^{\text{initial}} = 0.5$, which is maintained a constant in the simulations as there is no exchange exchange of water across the boundary (equivalent to a boundary condition of no outward flux of nucleoplasm $(\nabla\mu_n.\hat{\boldsymbol{n}}|_{\text{boundary}} = 0)$). As chromatin is confined to the nucleus, a no flux boundary condition of the order parameter $(\nabla\mu_d.\hat{\boldsymbol{n}}|_{\text{boundary}} = 0)$ ensures the conservation of epigenetic marks.

Spatial perturbation parameters: To mimic the spatial heterogeneities of the acetylation and methylation reactions, we add a Gaussian noise with a mean as the parameter values listed in Table SI and a 20% relative standard deviation. This gives us a chromatin domain distribution in agreement with the distribution of domain sizes observed in the experimental images. A similar Gaussian noise is also added to the strength of chromatin-lamina interactions to capture the heterogeneities in the anchoring of chromatin to the lamina. Lastly, we add a random uniform perturbation to the initial chromatin configuration to represent noise due to intrinsic heterogeneities present in the nucleus.

Table S2: Values of the parameters used in simulation.

	Parameter	Description	Value	Remarks
Initial Conditions	$\phi_n^{initial}$	Initial water (nucleoplasm) content in the nucleus	0.5	Estimated from experimental H2B density
	$\phi_d^{initial}$	Initial value of order parameter	0.1	The choice does not affect the simulations in presence of reactions
	σ_ϕ^{noise}	Range of uniform perturbation due to heterogeneities in initial conditions	0.01	

	Parameter	Meaning	Condition	Value	Remarks
Epigenetic Kinetics	$\tilde{\Gamma}_{me}$	Reaction rate of histone methylation (non-dimensionalised)		1.2	Estimated from H2B density in nuclei with transcription inhibited
	σ_{Γ}^{noise}	Variance in spatial distribution of reaction rates		0.2	Value large enough to obtain a spatial variation in domain sizes
Chromatin-lamina Interactions	$\tilde{V}_L$	Chromatin-lamina interaction strength (non-dimensionalised)		0.012	Parametric study performed to best approximate the LAD distribution in absence of transcription
	$\sigma_{\tilde{V}_L}^{noise}$	Variance in spatial distribution of $\tilde{V}_L$		$= \sigma_{\Gamma}^{noise}$	
Extrusion Kinetics	$\tilde{\Gamma}_a$	Rate of active chromatin extrusion	Control	0.03	Calibrated with experiments
			ActD treatment	0	Total inhibition of loop extrusion
			WAPLΔ	0.0075	Calibrated with experiments

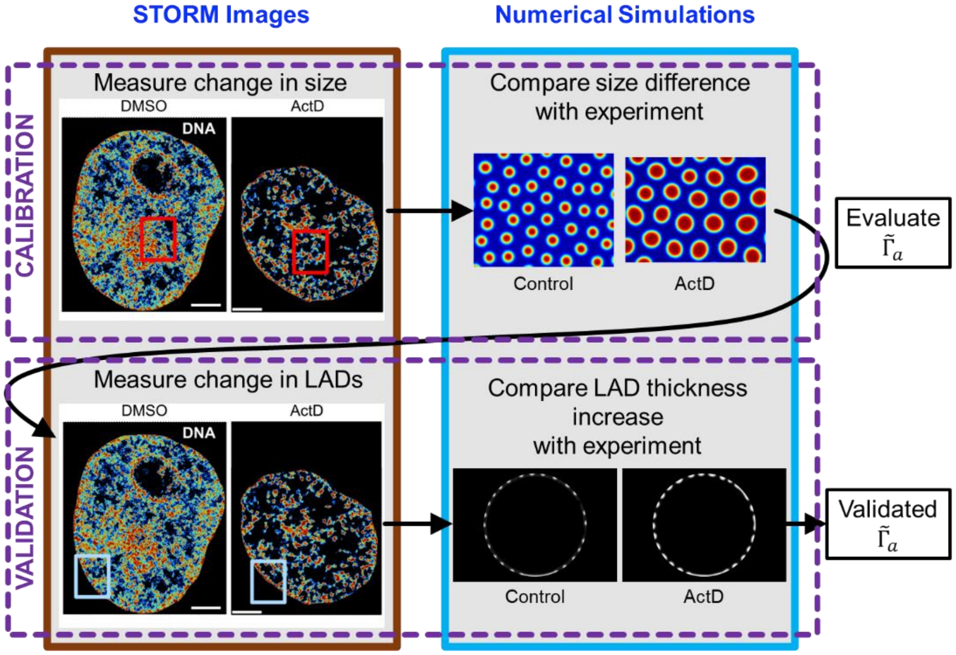

Figure S14: Methodology for calibration and validation of the extrusion rate parameter $\tilde{\Gamma}_a$ as described in Section 3.3. All scale bars 3 μm.

S9 Translating the model predictions into physical dimensions

We have used the rescaled model (see Section S1.5) to make non-dimensional quantitative predictions on the change in chromatin organization upon transcriptional perturbations via supercoiling driven loop extrusion. We can use rescaling parameters to obtain the physical interpretation of the timescales, length scales and the energy scales involved in our model. Table S3 below lists the values of the rescaling parameters, obtained from the published literature, and thus obtained values of time, length and energy scales.

Table S3: Experimental parameters are used to translate the non-dimensional model parameters into physical units

	**Parameter**	**Meaning**	**Order of magnitude**	**Reference**	**Remarks**

Reaction timescale	Γ_{ac}	Rate of histone acetylation reaction	$\sim 10^{-2}\ s^{-1}$	[27]	Calculated as reciprocal of timescale of reaction
	Γ_{me}	Rate of histone methylation reaction	$\sim 10^{-3} s^{-1}$	[28]	
Length-scale	D	Diffusivity of nucleosomes	$\sim 10^{-3} \mu m^2/s$	[29]	Diffusivity of nucleosomes calculated as slope of MSD curve
	ℓ_{RD}	Reaction-diffusion length scale	$\sim 300\ nm$	Calculated as $\ell_{RD} \sim \sqrt{D/\Gamma_{ac}}$	
	R_d^{ss}	Characteristic steady state size of heterochromatin domains	$\sim 100\ nm$ [29-34][1]	Calculated as $\sim \sqrt{\frac{3D}{\Gamma_{ac}} \frac{\Gamma_{me}}{\Gamma_{me}+\Gamma_{ac}}}$ (Eq S28)	
Diffusion timescale	Time for diffusion across inter-domain space		$\sim 5\ min$	Calculated as $\sim (2\ell_{RD})^2/D$ <br>(Note that this is similar to timescale of reactions)	
Energy scale	$\frac{k_B T}{\Omega}$	Energy scale of chromatin-chromatin interactions	$\sim 10^{-24} J/nm^3$	Calculated. Assuming nucleosomes interact with each other over a length scale of 10 nm	
	ℓ_{int}	Width of the smooth boundary of heterochromatic domains	$\sim 50\ nm$	Observed from ChromSTEM imaging (Figure 2c)	
	η	Penalty associated with formation of interfaces	$\sim 10^{-21} J/nm$	Calculated as $\ell_{int} = \sqrt{\frac{\eta\Omega}{k_B T}}$, (Section S1.5)	

S10 The qualitative predictions of the model are agnostic to extent of compaction of the heterochromatin phase

Our model predicts that chromatin exists in two phases which are not only transcriptionally distinct but are also differentially constituted. For instance, in our model, the euchromatin phase primarily consists of acetylated chromatin with a volume fraction $\phi_e^{EC} \sim 0.2 - 0.3$ and is water rich with volume fraction of nucleoplasm $\phi_n^{EC} \sim 0.7 - 0.8$. However, the euchromatin phase has no methylated heterochromatin content, i.e., $\phi_h^{EC} \sim 0$. On the other hand, the heterochromatin phase is rich in heterochromatic content $\phi_h^{HC} = \phi_h^{max}$, and has a very little water content, with $\phi_n^{HC} \sim 0.02$.

Note that the heterochromatin phase is only water-poor, and not water-free, i.e., $\phi_n^{HC} \neq 0$. Being water-poor, the heterochromatin phase is highly compacted, but not so compacted as to exclude water. This distinction becomes important because even the highly compacted heterochromatin phase includes multiple chromatin associated proteins such as HP1 [15] and certain histone methyltransferases [31].

The extent of chromatin compaction in the heterochromatin phase i.e., the maximum heterochromatin volume fraction $\phi_h^{HC} = \phi_h^{max}$ is chosen suitably to allow the presence of some euchromatin and nucleoplasm within the heterochromatic phase. We observe a water content of $\phi_h^{HC} \sim 0.04$. However, the exact values of these parameters, or the exact density (in Mbp/μm^3) of

[1] Note that the size mentioned is calculated theoretically and matches with the cited publications.

chromatin within the heterochromatin phase, could be obtained by molecular dynamic simulations at a resolution of nucleosomes, calibrated to single nucleosome live-cell super-resolution imaging (such as [29, 32]). Single molecule localization microscopy (SMLM), which allows visualization of individual nucleosome clusters combined with super-resolution fluctuation-assisted binding-activated localization microscopy (fBALM) has recently emerged as a promising avenue towards extracting the nucleus-wide 3D spatial DNA density (in Mbp/μm^3) [35]. Further, a combined computational and imaging framework involving polymer-based modeling at nucleosome level and super-resolution imaging, called Modeling immuno-OligoSTORM (MiOS) has previously been developed by Neguembor, et al. [36], which could potentially be used for observing such fine-scale features of DNA organization.

As an example, we modify the overall water content of the nucleus ($\bar{\phi}_n$) to modulate the extent of chromatin compaction and water content within the heterochromatin phase (ϕ_n^{HC}). The extent of chromatin compaction can also be modified in other ways, such as varying ϕ_h^{max}, but here we present one example to show that our model predictions are agnostic to the exact choice of these parameters. The chromatin organization predicted by the model is shown in Figure S15. Note that in Figure S15a the distribution of water in the nucleus is shown (as opposed to that of heterochromatin). However, the red region (with low water content) is still heterochromatin, and blue region (with high water content) is euchromatin. Further, we have annotated the exact value of local volume fraction of nucleoplasm within a heterochromatin domain, i.e., ϕ_n^{HC}. It can be seen that as the nucleus becomes water rich, the heterochromatin phase compaction varies. This is because changing the water content in the nucleus also changes the location of the energy well on the energy landscape.

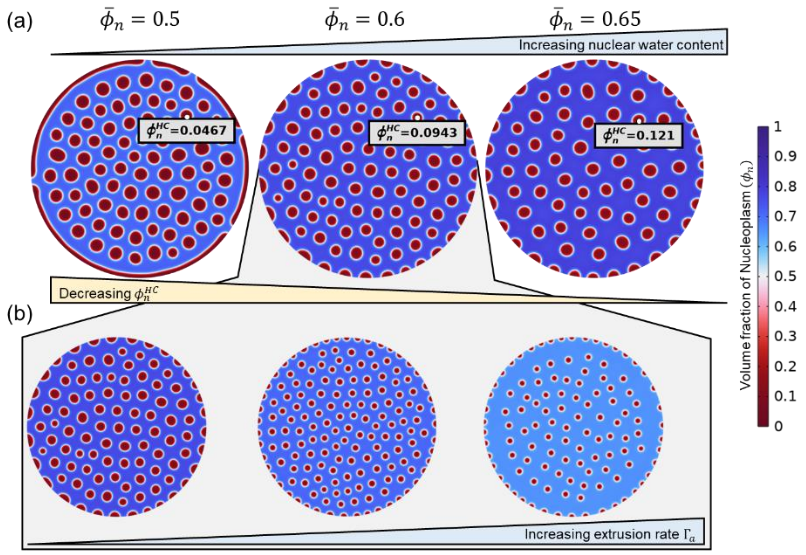

Figure S15: Chromatin organization predicted numerically as the overall water content in the nucleus varies. (a) As the nucleus becomes water rich, the heterochromatin phase becomes less compacted and contains a higher volume fraction of water. (b) For the case of $\bar{\phi}_n = 0.6$, as chromatin extrusion Γ_a increases, the heterochromatin domains become smaller.

For a specific case of $\bar{\phi}_n = 0.6$, we see that as the transcription increases the heterochromatin domain size scale in the interior and along the periphery reduces – in qualitative agreement with predictions reported in the main manuscript (Figure S15b). Thus, while choosing the exact level of chromatin compaction does quantitatively improve our prediction accuracy, it does not modify our model results, nor does it change the underlying physics of the model.

S11 Extending the model to incorporate multiple states of chromatin

The model proposed in this paper incorporates three nuclear constituents – nucleoplasm, heterochromatin and euchromatin. These constituents are mixed with each other and form two

stable phases – heterochromatin which is compacted and prominently methylated, and euchromatin which is loosely packed and prominently acetylated.

However, euchromatin as well as heterochromatin can have different subtypes depending on location (e.g., lysine site) and extent (e.g., mono-, di- or tri-) of methylation. These post-translational epigenetic variations can induce different functional properties to chromatin. As an example, H3K9me3 is expected to form a core of constitutive heterochromatin, which remains compacted at all stages of development in the cell, and all cell types [37, 38]. On the other hand, H3K27 in its trimethylated form H3K27me3 is a hallmark of Polycomb facultative heterochromatin, which can be reversibly switched between expressive (H3K27me1/2) or repressive (H3K27me3) forms [37, 38]. The formation of different classes of heterochromatin (constitutive and facultative) can involve different classes of epigenetic enzymes such as methyltransferase SUV39H1 and SUV39H2 for H3K9me3 or EZH2 for H3K27me3 [37].

As a simplified model to capture multiple states of chromatin, let us consider three nuclear constituents – euchromatin (which as a simplification is nucleoplasm rich by definition), constitutive heterochromatin and Polycomb-marked facultative heterochromatin. We consider that the three constituents can mix with different volume fractions to stably form three phases – each phase rich in one of each constituent. The free-energy landscape of this triphasic system can be defined as (analogous to Eq S1) [39],

$$W = \sum_{i=1,2,3} \left[A_i \phi_i^2 (\phi_i^{max} - \phi_i)^2 + \frac{\eta_i}{2} |\nabla \phi_i|^2 \right]$$

where ϕ_i represents the volume fraction of each constituent. The energy coefficients A_i have a scale of $k_B T/\Omega$. The energy landscape is represented on a ternary phase diagram as shown in

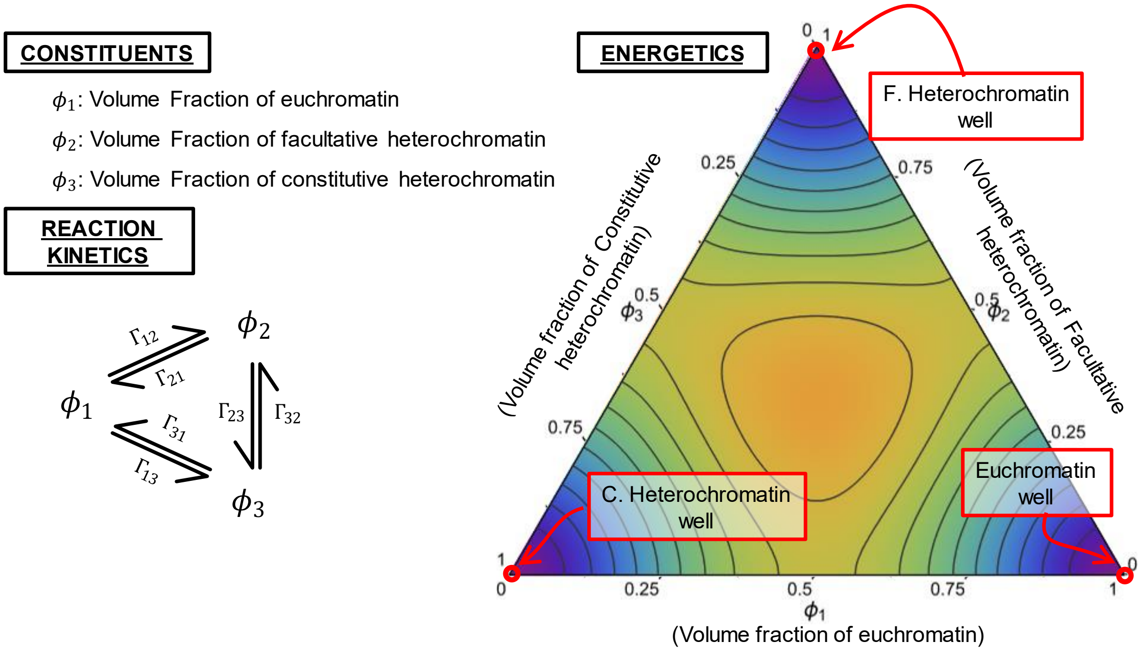

Figure S16: The key ingredients of a ternary phase field model to capture three stable phases of chromatin – euchromatin, facultative and constitutive heterochromatin.

Figure S16. Note that the energy landscape has three wells, or three minima, corresponding to the three stable phases. The location of these wells can be altered by appropriately choosing the

parameters ϕ_i^{max}. For the particular landscape in Figure S16, we have chosen $\phi_i^{max} = 1$. Further, several classes of epigenetic factors such as histone methyltransferases, acetyltransferases, demethylases and deacetylases allow a non-conservative interconversion between these phases, which are captured via the parameters Γ_{ij} denoting the rate of conversion of constituent i into constituent j, for $i, j = 1,2,3$, as shown in Figure S16.

The values of the kinetic parameters need to be appropriately chosen. For instance, it could be assumed that the facultative heterochromatin cannot be converted into constitutive heterochromatin directly ($\Gamma_{23} = 0$), although it may happen indirectly via $\Gamma_{21} \times \Gamma_{13}$ pathway.

Note that by incorporating three stable phases, we have now introduced C_2^3 energetic parameters, 3 interfacial energy parameters, and P_2^3 kinetic parameters. As the number of stable phases incorporated increases, the number of energetic and kinetic parameters increases combinatorically. Thus, from a modeling standpoint, it may make economic sense to choose specific stable forms of chromatin as required to reduce the number of parameters. The choice of which stable forms of chromatin are chosen to be modeled would depend on the specific phenomenon being modeled.

Another such choice of stable forms of chromatin could be euchromatin, heterochromatin, and an intermediate unmarked state of chromatin as shown in Figure S17. Note that if the intermediate state of chromatin is unstable and has a very short lifetime, the rate-determining steps are the demethylation and acetyltransferase reactions. Thus, with such an assumption, this multi-phase model simplifies to the model presented in the main manuscript.

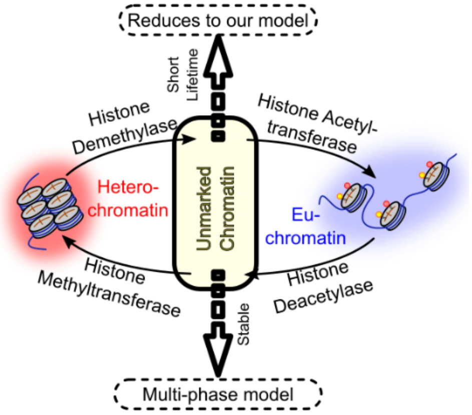

Figure S17: Another interpretation of multiple chromatin phases with an unmarked intermediate phase.

REFERENCES

1. Barton, L.J., A.A. Soshnev, and P.K. Geyer, *Networking in the nucleus: a spotlight on LEM-domain proteins.* Current opinion in cell biology, 2015. **34**: p. 1-8.
2. Luperchio, T.R., X. Wong, and K.L. Reddy, *Genome regulation at the peripheral zone: lamina associated domains in development and disease.* Current opinion in genetics & development, 2014. **25**: p. 50-61.
3. Zullo, J.M., et al., *DNA sequence-dependent compartmentalization and silencing of chromatin at the nuclear lamina.* Cell, 2012. **149**(7): p. 1474-1487.
4. Manzo, S.G., L. Dauban, and B. van Steensel, *Lamina-associated domains: Tethers and looseners.* Current Opinion in Cell Biology, 2022. **74**: p. 80-87.
5. Van Steensel, B. and A.S. Belmont, *Lamina-associated domains: links with chromosome architecture, heterochromatin, and gene repression.* Cell, 2017. **169**(5): p. 780-791.
6. Wong, X., et al., *Mapping the micro-proteome of the nuclear lamina and lamina-associated domains.* Life science alliance, 2021. **4**(5).
7. Cowie, J.M.G. and V. Arrighi, *Polymers: chemistry and physics of modern materials*. 2007: CRC press.
8. Zhu, J., et al., *Modelling and numerical simulation of phase separation in polymer modified bitumen by phase-field method.* Materials & design, 2016. **107**: p. 322-332.
9. Grosberg, A.Y. and J.-F. Joanny, *Nonequilibrium statistical mechanics of mixtures of particles in contact with different thermostats.* Physical Review E, 2015. **92**(3): p. 032118.

10. Chen, L.-Q., *Phase-field models for microstructure evolution.* Annual review of materials research, 2002. **32**(1): p. 113-140.
11. Kim, K., et al., *First-principles/Phase-field modeling of θ' precipitation in Al-Cu alloys.* Acta Materialia, 2017. **140**: p. 344-354.
12. Ramanarayan, H. and T. Abinandanan, *Phase field study of grain boundary effects on spinodal decomposition.* Acta materialia, 2003. **51**(16): p. 4761-4772.
13. Tanaka, H., *Viscoelastic phase separation.* Journal of Physics: Condensed Matter, 2000. **12**(15): p. R207.
14. Sanulli, S. and G.J. Narlikar, *Liquid-like interactions in heterochromatin: Implications for mechanism and regulation.* Current opinion in cell biology, 2020. **64**: p. 90-96.
15. Sanulli, S., et al., *HP1 reshapes nucleosome core to promote phase separation of heterochromatin.* Nature, 2019. **575**(7782): p. 390-394.
16. Bian, C., et al., *Sgf29 binds histone H3K4me2/3 and is required for SAGA complex recruitment and histone H3 acetylation.* The EMBO journal, 2011. **30**(14): p. 2829-2842.
17. Andronov, L., et al., *ClusterViSu, a method for clustering of protein complexes by Voronoi tessellation in super-resolution microscopy.* Scientific reports, 2016. **6**(1): p. 1-9.
18. Ester, M., et al. *A density-based algorithm for discovering clusters in large spatial databases with noise*. in *kdd*. 1996.
19. Ulianov, S.V., et al., *Nuclear lamina integrity is required for proper spatial organization of chromatin in Drosophila.* Nature communications, 2019. **10**(1): p. 1-11.
20. Ou, H.D., et al., *ChromEMT: Visualizing 3D chromatin structure and compaction in interphase and mitotic cells.* Science, 2017. **357**(6349): p. eaag0025.
21. Kremer, J.R., D.N. Mastronarde, and J.R. McIntosh, *Computer visualization of three-dimensional image data using IMOD.* Journal of structural biology, 1996. **116**(1): p. 71-76.
22. Gürsoy, D., et al., *TomoPy: a framework for the analysis of synchrotron tomographic data.* Journal of synchrotron radiation, 2014. **21**(5): p. 1188-1193.
23. Li, Y., et al., *Analysis of three-dimensional chromatin packing domains by chromatin scanning transmission electron microscopy (ChromSTEM).* Scientific reports, 2022. **12**(1): p. 1-15.
24. Goedhart, J., *SuperPlotsOfData—a web app for the transparent display and quantitative comparison of continuous data from different conditions.* Molecular biology of the cell, 2021. **32**(6): p. 470-474.
25. Eid, A., et al., *Characterizing chromatin packing scaling in whole nuclei using interferometric microscopy.* Optics letters, 2020. **45**(17): p. 4810-4813.
26. Heo, S.-J., et al., *Aberrant chromatin reorganization in cells from diseased fibrous connective tissue in response to altered chemomechanical cues.* Nature Biomedical Engineering, 2022: p. 1-15.
27. Waterborg, J.H., *Dynamics of histone acetylation in vivo. A function for acetylation turnover?* Biochemistry and cell biology, 2002. **80**(3): p. 363-378.
28. Haws, S.A., et al., *Intrinsic catalytic properties of histone H3 lysine-9 methyltransferases preserve monomethylation levels under low S-adenosylmethionine.* Journal of Biological Chemistry, 2023: p. 104938.
29. Nozaki, T., et al., *Condensed but liquid-like domain organization of active chromatin regions in living human cells.* Science Advances, 2023. **9**(14): p. eadf1488.
30. Markaki, Y., et al. *Functional nuclear organization of transcription and DNA replication a topographical marriage between chromatin domains and the interchromatin compartment*. in *Cold Spring Harbor symposia on quantitative biology*. 2010. Cold Spring Harbor Laboratory Press.

31. Miron, E., et al., *Chromatin arranges in chains of mesoscale domains with nanoscale functional topography independent of cohesin.* Science advances, 2020. **6**(39): p. eaba8811.
32. Nozaki, T., et al., *Dynamic organization of chromatin domains revealed by super-resolution live-cell imaging.* Molecular cell, 2017. **67**(2): p. 282-293. e7.
33. Gómez-García, P.A., et al., *Mesoscale modeling and single-nucleosome tracking reveal remodeling of clutch folding and dynamics in stem cell differentiation.* Cell reports, 2021. **34**(2).
34. Ricci, M.A., et al., *Chromatin fibers are formed by heterogeneous groups of nucleosomes in vivo.* Cell, 2015. **160**(6): p. 1145-1158.
35. Gelléri, M., et al., *True-to-scale DNA-density maps correlate with major accessibility differences between active and inactive chromatin.* Cell Reports, 2023. **42**(6).
36. Neguembor, M.V., et al., *MiOS, an integrated imaging and computational strategy to model gene folding with nucleosome resolution.* Nature Structural & Molecular Biology, 2022. **29**(10): p. 1011-1023.
37. Allshire, R.C. and H.D. Madhani, *Ten principles of heterochromatin formation and function.* Nature reviews Molecular cell biology, 2018. **19**(4): p. 229-244.
38. Bell, O., et al., *Heterochromatin definition and function.* Nature Reviews Molecular Cell Biology, 2023: p. 1-4.
39. Boyer, F. and C. Lapuerta, *Study of a three component Cahn-Hilliard flow model.* ESAIM: Mathematical Modelling and Numerical Analysis, 2006. **40**(4): p. 653-687.
