# Supplementary material for: Active transcription and epigenetic reactions synergistically regulate meso-scale genomic organization
Source: Nat Commun. 2024 May 21;15:4338. doi: 10.1038/s41467-024-48698-z (PMC11109243; doi:10.1038/s41467-024-48698-z)
Supplement: Supplementary file 3 — Reporting Summary [file 41467_2024_48698_MOESM3_ESM.pdf]

Corresponding author(s): Vivek B. Shenoy

Last updated by author(s): Apr 23, 2024

Reporting Summary

Nature Portfolio wishes to improve the reproducibility of the work that we publish. This form provides structure for consistency and transparency in reporting. For further information on Nature Portfolio policies, see our Editorial Policies and the Editorial Policy Checklist.

Statistics

For all statistical analyses, confirm that the following items are present in the figure legend, table legend, main text, or Methods section.

n/a	Confirmed	
☐	☒	The exact sample size (*n*) for each experimental group/condition, given as a discrete number and unit of measurement
☐	☒	A statement on whether measurements were taken from distinct samples or whether the same sample was measured repeatedly
☐	☒	The statistical test(s) used AND whether they are one- or two-sided<br>*Only common tests should be described solely by name; describe more complex techniques in the Methods section.*
☒	☐	A description of all covariates tested
☒	☐	A description of any assumptions or corrections, such as tests of normality and adjustment for multiple comparisons
☐	☒	A full description of the statistical parameters including central tendency (e.g. means) or other basic estimates (e.g. regression coefficient) AND variation (e.g. standard deviation) or associated estimates of uncertainty (e.g. confidence intervals)
☐	☒	For null hypothesis testing, the test statistic (e.g. *F*, *t*, *r*) with confidence intervals, effect sizes, degrees of freedom and *P* value noted<br>*Give P values as exact values whenever suitable.*
☒	☐	For Bayesian analysis, information on the choice of priors and Markov chain Monte Carlo settings
☒	☐	For hierarchical and complex designs, identification of the appropriate level for tests and full reporting of outcomes
☒	☐	Estimates of effect sizes (e.g. Cohen's *d*, Pearson's *r*), indicating how they were calculated

Our web collection on statistics for biologists contains articles on many of the points above.

Software and code

Policy information about availability of computer code

Data collection	Collection of STORM image datasets is originally described in Neguembor et al., Mol Cell 2021.<br><br>ChromSTEM tomograms and PWS images were acquired using methods described previously (Li et al., Scientific Reports, 2022; Li et al., Science Advances, 2021). The Python module for data acquisition is publicly available on GitHub at: https://github.com/BackmanLab/PWSpy
Data analysis	STORM images were analyzed using a custom written MATLAB code (made publicly available and citable on GitHub at: https://github.com/ShenoyLab/STORM_Analysis) to obtain the sizes of the heterochromatin domains for HeLa cell lines.<br><br>ChromSTEM tomograms and PWS images were analyzed using methods described previously for evaluating domain properties like scaling, size, and density (Li et al., Scientific Reports, 2022; Li et al., Science Advances, 2021). The Python module for PWS acquisition and analysis is also publicly available on GitHub at: https://github.com/BackmanLab/PWSpy<br><br>PDEs describing the mathematical model were solved using COMSOL, a commercially available software, using weak form PDE module.<br><br>Softwares used for analysis: MATLAB R2022a (for 3D ChromSTEM domain analysis, STORM domain analysis); Image J/FIJI (for domain center identification PWS); Microsoft Excel (for statistical analysis).

For manuscripts utilizing custom algorithms or software that are central to the research but not yet described in published literature, software must be made available to editors and reviewers. We strongly encourage code deposition in a community repository (e.g. GitHub). See the Nature Portfolio guidelines for submitting code & software for further information.

Data

Policy information about availability of data

All manuscripts must include a data availability statement. This statement should provide the following information, where applicable:

- Accession codes, unique identifiers, or web links for publicly available datasets
- A description of any restrictions on data availability
- For clinical datasets or third party data, please ensure that the statement adheres to our policy

The authors declare that the data supporting the findings of this study are available within the paper and its supplementary information files. The data generated in this study are provided in the Source Data file.

Research involving human participants, their data, or biological material

Policy information about studies with human participants or human data. See also policy information about sex, gender (identity/presentation), and sexual orientation and race, ethnicity and racism.

Reporting on sex and gender	N/A
Reporting on race, ethnicity, or other socially relevant groupings	N/A
Population characteristics	N/A
Recruitment	N/A
Ethics oversight	N/A

Note that full information on the approval of the study protocol must also be provided in the manuscript.

Field-specific reporting

Please select the one below that is the best fit for your research. If you are not sure, read the appropriate sections before making your selection.

☒ Life sciences ☐ Behavioural & social sciences ☐ Ecological, evolutionary & environmental sciences

For a reference copy of the document with all sections, see nature.com/documents/nr-reporting-summary-flat.pdf

Life sciences study design

All studies must disclose on these points even when the disclosure is negative.

Sample size	STORM image datasets were originally described in Neguembor et al., Mol Cell 2021 in which no sample size calculation has been performed before sample collection. Due to the low throughput nature of STORM imaging technique, as many images as possible were collected in multiple fields of view and chromatin compaction observations were verified by performing multiple types of imaging (histone modifications, DNA, cohesin) by STORM and with orthogonal techniques allowing larger dataset collection such confocal imaging and live cell imaging.<br><br>ChromSTEM was performed on a 2um x 2um x 100nm volume from nuclei from 1 control and 1 Actinomycin D treated cell. The sample size is enough to cover ~50 domains from each condition for a comparison. Due to the low throughput nature of the technique no sample size calculation was performed in that case.<br><br>For PWS live cell imaging, the number of cells analyzed was estimated to achieve a 95% confidence level given the expected difference between the groups being tested.
Data exclusions	No imaging data were excluded from the analysis. All collected datasets were utilized for the subsequent analysis steps.
Replication	All live cell PWS experiments were carried out across 5 different cell lines to confirm similar results are acquired.<br><br>Findings from high-resolution electron microscopy (ChromSTEM) were not replicated. High Resolution Electron Tomography is not cost-efficient for repeated studies. However, the change in chromatin structure observed in ChromSTEM is confirmed with PWS on n>60 nuclei over 5 different cell lines.
Randomization	All samples were randomly assigned to each group.
Blinding	Blinding was not possible during data acquisition because specific treatments were added by the investigator to each group before imaging. Investigators were blinded to imaging data analysis.

Reporting for specific materials, systems and methods

We require information from authors about some types of materials, experimental systems and methods used in many studies. Here, indicate whether each material, system or method listed is relevant to your study. If you are not sure if a list item applies to your research, read the appropriate section before selecting a response.

Materials & experimental systems

n/a	Involved in the study
☒	☐ Antibodies
☐	☒ Eukaryotic cell lines
☒	☐ Palaeontology and archaeology
☒	☐ Animals and other organisms
☒	☐ Clinical data
☒	☐ Dual use research of concern
☒	☐ Plants

Methods

n/a	Involved in the study
☒	☐ ChIP-seq
☒	☐ Flow cytometry
☒	☐ MRI-based neuroimaging

Eukaryotic cell lines

Policy information about cell lines and Sex and Gender in Research

Cell line source(s)	BJ Fibroblasts, A549, Hela, U2OS, and HCT116 were purchased from the American Type Culture Collection (ATCC).
Authentication	Authentication of the cell lines was performed by ATCC Universal Mycoplasma Detection Kit using PCR-based assay with species-specific primers
Mycoplasma contamination	All of the cell lines used for these studies tested negative for mycoplasma using the ATCC Universal Mycoplasma Detection kit
Commonly misidentified lines (See ICLAC register)	No commonly misidentified cell lines were used.

Plants

Seed stocks	*Report on the source of all seed stocks or other plant material used. If applicable, state the seed stock centre and catalogue number. If plant specimens were collected from the field, describe the collection location, date and sampling procedures.*
Novel plant genotypes	*Describe the methods by which all novel plant genotypes were produced. This includes those generated by transgenic approaches, gene editing, chemical/radiation-based mutagenesis and hybridization. For transgenic lines, describe the transformation method, the number of independent lines analyzed and the generation upon which experiments were performed. For gene-edited lines, describe the editor used, the endogenous sequence targeted for editing, the targeting guide RNA sequence (if applicable) and how the editor was applied.*
Authentication	*Describe any authentication procedures for each seed stock used or novel genotype generated. Describe any experiments used to assess the effect of a mutation and, where applicable, how potential secondary effects (e.g. second site T-DNA insertions, mosiacism, off-target gene editing) were examined.*
